# Field phenotyping for African crops: overview and perspectives

**DOI:** 10.3389/fpls.2023.1219673

**Published:** 2023-10-04

**Authors:** Daniel K. Cudjoe, Nicolas Virlet, March Castle, Andrew B. Riche, Manal Mhada, Toby W. Waine, Fady Mohareb, Malcolm J. Hawkesford

**Affiliations:** ^1^ Sustainable Soils and Crops, Rothamsted Research, Harpenden, United Kingdom; ^2^ School of Water, Energy and Environment, Cranfield University, Cranfield, Bedfordshire, United Kingdom; ^3^ AgroBiosciences Department, Mohammed VI Polytechnic University (UM6P), Benguérir, Morocco

**Keywords:** African crops, phenotypes, field phenotyping, high-throughput phenotyping, phenotyping infrastructures, low-cost phenotyping, African agriculture, precision agriculture

## Abstract

Improvements in crop productivity are required to meet the dietary demands of the rapidly-increasing African population. The development of key staple crop cultivars that are high-yielding and resilient to biotic and abiotic stresses is essential. To contribute to this objective, high-throughput plant phenotyping approaches are important enablers for the African plant science community to measure complex quantitative phenotypes and to establish the genetic basis of agriculturally relevant traits. These advances will facilitate the screening of germplasm for optimum performance and adaptation to low-input agriculture and resource-constrained environments. Increasing the capacity to investigate plant function and structure through non-invasive technologies is an effective strategy to aid plant breeding and additionally may contribute to precision agriculture. However, despite the significant global advances in basic knowledge and sensor technology for plant phenotyping, Africa still lags behind in the development and implementation of these systems due to several practical, financial, geographical and political barriers. Currently, field phenotyping is mostly carried out by manual methods that are prone to error, costly, labor-intensive and may come with adverse economic implications. Therefore, improvements in advanced field phenotyping capabilities and appropriate implementation are key factors for success in modern breeding and agricultural monitoring. In this review, we provide an overview of the current state of field phenotyping and the challenges limiting its implementation in some African countries. We suggest that the lack of appropriate field phenotyping infrastructures is impeding the development of improved crop cultivars and will have a detrimental impact on the agricultural sector and on food security. We highlight the prospects for integrating emerging and advanced low-cost phenotyping technologies into breeding protocols and characterizing crop responses to environmental challenges in field experimentation. Finally, we explore strategies for overcoming the barriers and maximizing the full potential of emerging field phenotyping technologies in African agriculture. This review paper will open new windows and provide new perspectives for breeders and the entire plant science community in Africa.

## 1 Introduction

The global demand for food is projected to increase in the coming decades, driven by population growth, climate change, pandemics, shifts in food consumption and biofuel use (Tilman et al., 2011; Godfray and Robinson, 2015; van Dijk et al., 2021). Ensuring that crop production is sufficient to meet future goals is a challenge for plant and agricultural sciences.

In Africa, agricultural crops provide food and income for smallholder farmers and consumers. Despite the huge agricultural potential, agricultural productivity in African countries continues to remain the lowest in the world (Bjornlund et al., 2020). Many studies have indicated that yields of several important staple crops may be stagnating or even declining across the continent (Roudier et al., 2011; Knox et al., 2012; Ray et al., 2012; Parkes et al., 2018). This is the case for key staple crops such as maize, rice, wheat, millet, sorghum, cowpea, cassava and yam, which together account for a large portion of the population’s diet. Therefore, food supply systems would be negatively affected if yield gains in these crops continue to slow due to environmental stresses and production constraints.

Addressing food security in Africa is a vast challenge that needs to be tackled in many complementary directions. Infrastructure development adapted to local needs, good farming practices, management, and political will are some of the major axes of development for food security. Improving crop performance and tolerance/resistance to biotic and abiotic conditions is the challenge facing the scientific community and innovative methods are needed.

Advanced field phenotyping, e.g. using digital approaches, has developed substantially over the past decade and provides means for real-time monitoring of response to environmental stresses and nutrition, and aids unravelling the relationships between yield and complex genotypic traits. The identification of genotypes with superior traits of agricultural interest remains one of the major targets for the genetic improvement of crops (Varshney et al., 2021).

The genomes of many agricultural crops such as rice (Matsumoto et al., 2005), sorghum (Paterson et al., 2009), maize (Schnable et al., 2009), soybean (Schmutz et al., 2010) and recently wheat (Appels et al., 2018) have been sequenced. However, the advances made in genomic approaches such as maker-assisted selection and high-throughput sequencing (Crossa et al., 2017; Scheben et al., 2018) are yet to be complemented with accurate field phenotyping methods (Minervini et al., 2015). Most of the traits of agronomic relevance (e.g., yield) are complex, and quantitative, requiring tools for their phenotypic assessment in the field (Reynolds et al., 2020). Furthermore, open field rather than controlled environment measurements are more likely to be useful in identifying genotypes that will perform better in farming practice, especially when large plots that mimic real farm conditions (i.e., environmental and management conditions) are employed (Rebetzke et al., 2014).

In addition, precision agriculture (PA) is becoming increasingly important in today’s technologically advanced world (Langemeier and Boehlje, 2021; Gobezie and Biswas, 2023) and PA remains one of the cardinal principles of field phenotyping. The PA farming management concept relies on modern digital techniques to monitor and optimize agricultural production processes to improve crop performance (Hedley, 2015; Gokool et al., 2023). Despite PA’s contributions to sustainable agriculture, its use in resource-constrained smallholder farming environments, particularly in Sub-Saharan Africa (SSA), has been very limited (Gobezie and Biswas, 2023). Recent developments in sensor technologies, machine vision, and higher-resolution digital cameras, in tandem with advanced data processing power and other portable tools have paved the way for high-throughput plant phenotyping in the field to benefit crop breeding programs (Deery et al., 2014; Zhang et al., 2016; Araus et al., 2022; Ahmed et al., 2023). From the field phenotyping perspective, these emerging technologies are enabling automated intensive data collection and increasing the ability to investigate plant function and structure through non-invasive methods with high accuracy. Such field phenotyping methods will aid crop improvement efforts to meet the expected demand for food and agricultural products in the future.

The development and application of these high-throughput tools for field phenotyping are currently focused on the main staple crops grown in the most developed agricultural regions. Over the decades, breeders and agronomists in Africa have used traditional phenotyping based on manual methods either for selecting traits or for improving yields through changes in agronomic practices (Iizumi and Sakai, 2020). However, traditional phenotyping in breeding is time-consuming, laborious and data collection is insufficient to fulfil the needs of plant breeders which impedes breeding progress. Therefore, further advances in phenotyping methods and appropriate implementation are required to increase the effectiveness of selection in breeding programs, speed up genetic gains, reduce costs and enable monitoring of plant status more efficiently than is currently feasible. The sophistication and cost of current plant phenotyping equipment (Reynolds et al., 2019) have restricted them from being widely applied in the developing world and especially in Africa. Additionally, insufficient technical, operational, regulatory restrictions and conceptual capacity in the plant science community have further limited implementation. Therefore, it is timely to begin to apply these technologies more widely, both geographically and with respect to target crops in Africa. Affordable high-throughput phenotyping aims to achieve reasonably priced solutions for all the components comprising the phenotyping pipeline which will promote their adoption for the breeding of African crops (Whalen and Yuhas, 2019; Bongomin et al., 2022).

Few studies have covered the use of modern field phenotyping approaches employing remote sensing in Africa (e.g., Mutanga et al., 2016; Chivasa et al., 2017; Buchaillot et al., 2019; Bongomin et al., 2022; Kassim et al., 2022). For instance, Bongomin et al. (2022) recently reviewed the status of field phenotyping in Uganda with focus on the application of drones and image analytics.

In this review, we provide a background on African agriculture and cover the concept of digital field phenotyping, focused on traits that may be measured by emerging technologies and which could be applicable to African crops. The current developments of field phenotyping in Africa, including initiatives, implementation challenges and prospects are comprehensively reviewed. We observed that the lack of suitable field phenotyping infrastructures and approaches using digital technologies is limiting the development of improved crop cultivars and will negatively affect the agricultural industry and food security in Africa. We emphasize the potential for incorporating cutting-edge, low-cost phenotyping tools (i.e., portable field sensors, UAVs) into breeding schemes and for identifying agricultural crop responses to environmental constraints through field experimentation. Finally, we consider policy directions for tackling the implementation challenges (i.e., practical, financial, geographical and political) of digital field phenotyping and realizing the full potential of available field phenotyping resources (i.e., technologies, tools and know-how) appropriate for African crops.

## 2 African crops and the challenges to production

African countries are important producers of major crops with diverse agro-climatic and ecological conditions, and cultural diversity (Leakey et al., 2022). Sub-Saharan West Africa is composed of a wide variety of ecosystems and an equally high number of production systems (https://www.fao.org/3/AC349E/ac349e04.htm). Generally, crop production is concentrated in areas with a favourable combination of agro-bioclimatic conditions. In the Sahelian zone, cereals such as millet and sorghum are the predominant crops with annual rainfall (200-600 mm), transitioning to maize, groundnuts and cowpeas farther south in the Sudanian savannah zone (the so-called “Middle Belt”). These food crops are among the top five harvested crops in the Sahelian countries – Burkina Faso, Senegal, Mauritania, Mali, Chad and Niger. According to FAOSTAT (2018a) data, maize is the major essential staple food in sub-Saharan Africa, accounting for nearly 20% of total calorie intake. The same source indicates that in Sub-Saharan West Africa, millet and sorghum account for roughly 64% of total cereal production. Across the rainy forests of the Guinean zone (1200-2200 mm of rainfall per year) crops are predominantly root and tuber crops such as cassava and yams which are mostly cultivated in Ghana, Nigeria, Côte d’Ivoire and Sierra Leone. Yam is the second most important crop in Africa in terms of production after cassava (FAOSTAT, 2018a). Rice, on the other hand, is one of the most widely harvested crops in this humid zone, ranking first in Guinea, Liberia and Sierra Leone in terms of area harvested (Soullier et al., 2020; Duvallet et al., 2021).

Crop production in West Africa is mostly rainfed and crop production is vulnerable to climate change, which manifests itself in unpredictably high temperatures and erratic rainfall patterns (Sultan and Gaetani, 2016; Affoh et al., 2022). The five principal crops in West Africa in terms of harvested area (in millions of hectares per year on average in the last decade) are cassava (81), maize (19), millet (10), sorghum (12), yam (57) (FAOSTAT, 2022). Major cash crops are cocoa, coffee and cotton. Declining soil fertility and unpredictable climate change impacts (among other factors) have made it difficult to maintain the yields of these major crops (Shimeles et al., 2018). Over the last three decades, the agricultural sector in West Africa has been characterized by strong production growth in some major staple crops culminating in increased production volumes for both domestic and export markets (Blein et al., 2008; FAO, 2015). Similarly to West Africa, Central Africa’s principal food crops include cassava, peanuts, sorghum, millet, maize, sesame and plantains. Additionally major cash crops for export include cotton, coffee and tobacco (Ochieng et al., 2020).

In Northern Africa, particularly Morocco, crop production is regionally diverse owing to different climatic conditions, agro-ecological zones, land-crop tenure and farming systems (Ouraich and Tyner, 2018). This geographical diversity results in varied agriculture, with crops ranging from cereals and vegetables to fruits and nuts, grains, legumes, etc., that contribute significantly towards the country’s agricultural sustainability and food security. Cereal production accounts for 65% of cultivable agricultural areas (Ouraich and Tyner, 2018). Most cereal production occurs under rainfed conditions. As a result, productivity performance is influenced by precipitation levels. For instance, 7.3 million tonnes of wheat were produced in 2018 making it the 20th largest producer in the world and 2.8 million tonnes of barley being the 15th largest producer in the world (FAOSTAT, 2018b). However, drought is a persistent threat to crop production especially the lowlands where cereals are grown are particularly at risk because of the wide variations in annual precipitation (Verner et al., 2018; Meliho et al., 2020). In recent years, quinoa has sparked particular attention in Morocco (Choukr-Allah et al., 2016; Hirich et al., 2021). It remains one of the most nutrient-dense crops and is recognized as a ‘Superf Food’ due to its nutritional benefits. Thus, Morocco is one of the few North African countries capable of achieving self-sufficiency in food production (Saidi and Diouri, 2017).

Grains and cereals (e.g., maize, wheat, barley, oats and sorghum) are South Africa’s most important crops occupying more than 60% of the acreage under cultivation (FAO, 2022). Together, these crops account for one of the largest agricultural industries contributing more than 30% to the total gross value of agricultural production (FAO, 2022). Maize, the country’s most important crop and largest locally produced field crop, is a dietary staple supplying most of the carbohydrate needs, a source of livestock feed and is an export crop (Epule et al., 2022).

The country has emerged as the largest maize producer and exporter in the Southern African Development Community (SADC) region and Africa as a whole (Fisher et al., 2015; FAO, 2022). According to the FAO, 2022, in 2021 South Africa produced 17 million metric tonnes of maize, making it the 9th largest producer in the world. Moreover, it produced 2.6 million metric tonnes of potato and 2.3 million metric tonnes of wheat. Largely, South Africa has a semi-arid climate characterized by summer and winter rainfall seasons. Unpredictable weather conditions due to climate change have a severe impact on maize and wheat production which accounts for more than 36% of the total value of field crops (Bradshaw et al., 2022).

**Figure 1:**
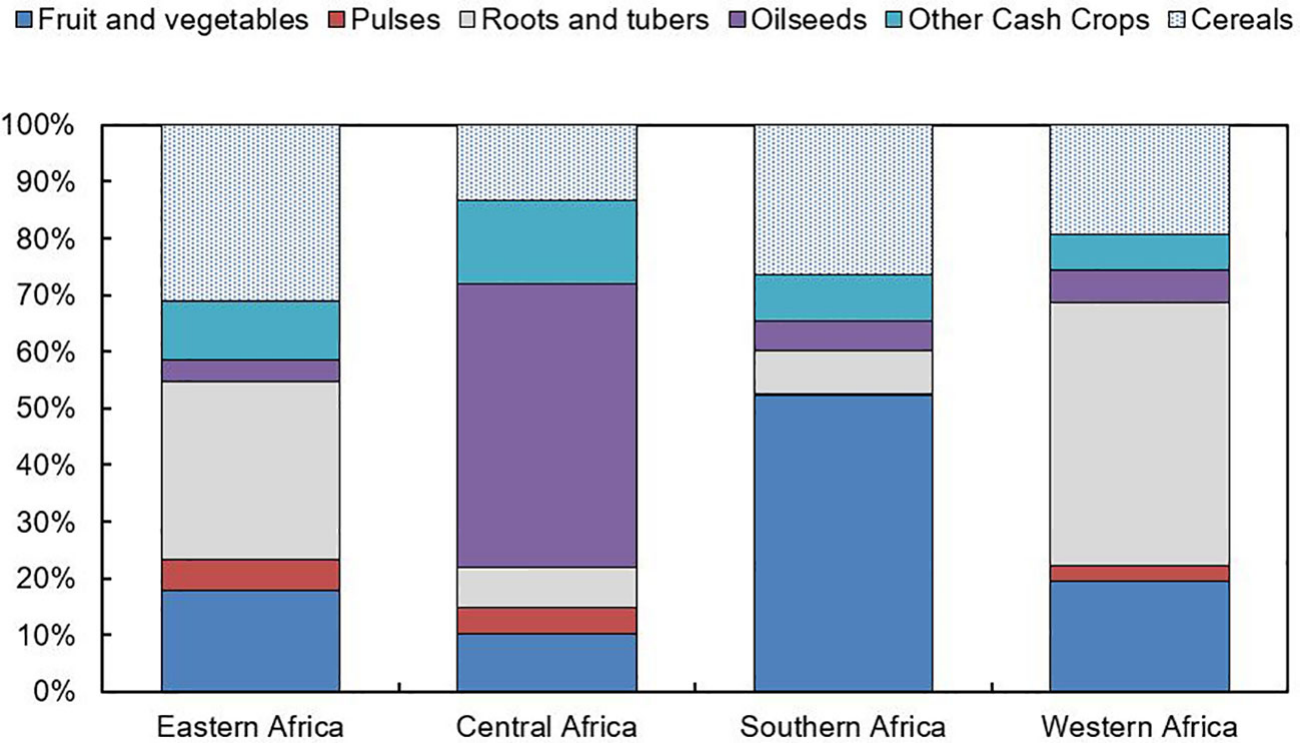
Major crop distribution in Sub-Saharan African region based on average production values between 2011-13. Adapted from FAOSTAT (2016). FAO, http://faostat3.fao.org/.

Smallholder farmers dominate agriculture in East African countries, contributing up to 90% of total agricultural production (Salami et al., 2010; Livingston et al., 2011). A cereal‐legume mixed cropping pattern is the dominant system that includes maize, millet, sorghum and wheat (Van Duivenbooden et al., 2000). Over 40% of the region is covered by the maize mixed cropping system, which is followed by the pastoral (14%), root crop (12%) and cereal-root crop mixed system (11%) (Adhikari et al., 2015). Teff is a significant crop in the Ethiopian highlands, while other significant crops in the area include cassava, bananas and rice. The mixed cropping system in East Africa is based on millet in the drier regions and on maize and cassava in the humid regions (Adhikari et al., 2015). The main cash crops in most of the East African countries in SSA are coffee, tea, cotton, tobacco and sugarcane. Rainfall variability negatively impact on crop production in East African countries (Palmer et al., 2023). Generally, the major challenges to crop production in Africa are unproductive soils, pests and diseases, drought, and poor crop management (Tadele, 2017). The distribution of major crops in each sub-region except Northern Africa is summarized in **Figure 1**.

## 3 Digital and image-based field phenotyping

Experiments with repeated trials in diverse environments are often necessary to screen plants for desirable traits. This becomes problematic when there is the need to screen a large panel of genotypes for valuable traits (i.e., yield potential or abiotic and biotic stress tolerance) to assess genotype, environment, and management (G × E× M) interactions (Araus and Cairns, 2014). Over the years, the measurement of individual plants in controlled conditions has dominated most of the phenotyping research. However, controlled environments often do not accurately mimic plant growth and development in field conditions (White et al., 2012). Field phenotyping is becoming more widely recognized as the approach that gives the most accurate representation of traits in real-world cropping systems (Tariq et al., 2020). Thus, field phenotyping is an important component of crop improvement to assess how the genotype, the environment, and their interaction (G × E) influence quantitative traits in a complex and dynamic manner (Fiorani and Schurr, 2013; Araus and Cairns, 2014; Neilson et al., 2015). Furthermore, field phenotyping is employed to discover novel traits, identify new germplasm carrying relevant but complex traits for breeding, and for testing proof of concept to validate traits (Watt et al., 2020). Traditionally, destructive sampling has been used to quantify certain observable plant traits, including laboratory analysis to characterize phenotypes based on their genetic and physiological functions. Digital phenotyping approaches seek to reduce this need (Tripodi et al., 2022; Virlet et al., 2022).

Different measurement approaches including novel technologies such as non-invasive imaging, robotics and sensor positioning systems have been incorporated in well-designed field phenotyping installations for high-throughput phenotyping (e.g., Araus and Cairns, 2014; Kirchgessner et al., 2017; Shakoor et al., 2017; Virlet et al., 2017; Pieruschka and Schurr, 2022). These significant strides in field phenotyping have fostered a major international collaborative effort directed toward data and protocol standardization (Pieruschka and Schurr, 2019; Lorence and Jimenez, 2022). The appeal of these platforms is the increased throughput and objectivity in data collection compared to traditional field approaches.

**Figure 2:**
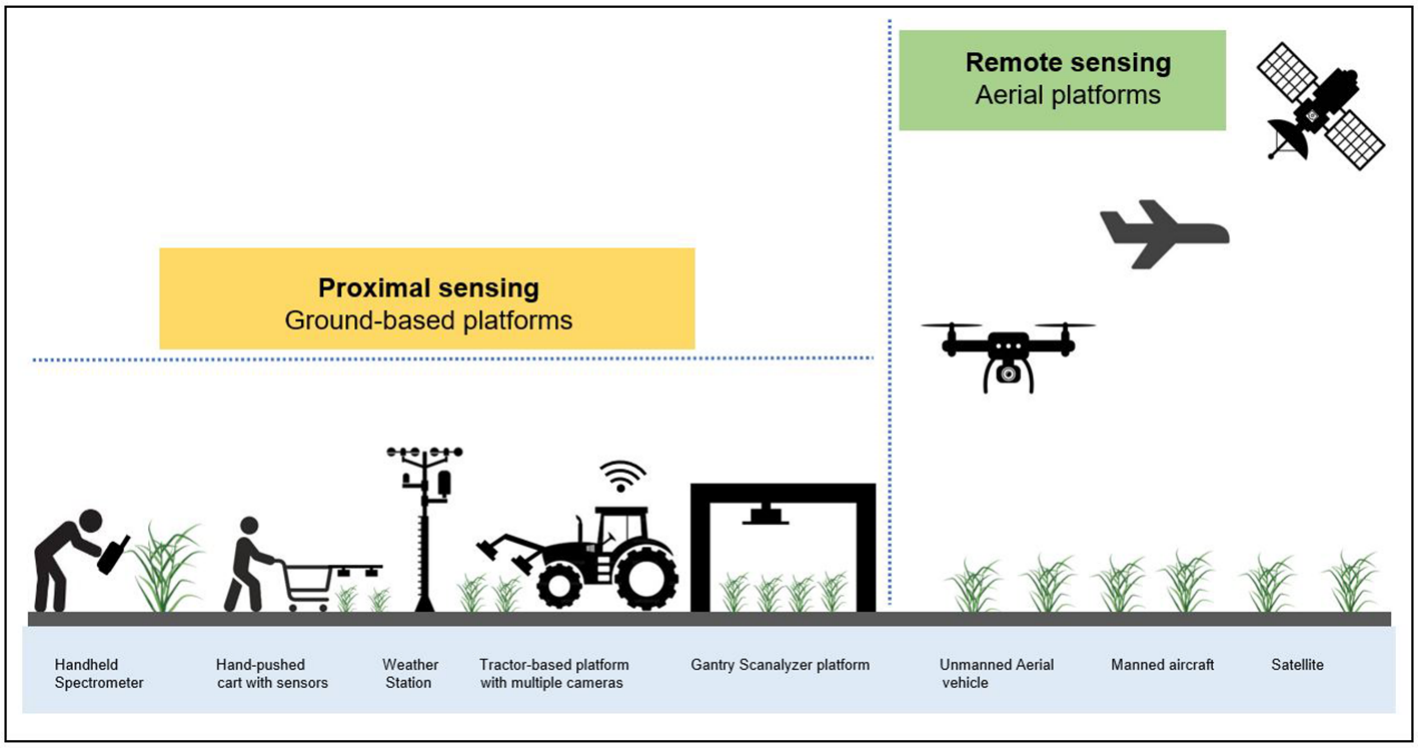
Overview of the most common field phenotyping systems and approaches at proximal and remote sensing scales. The proximal sensing approach is based on ground-based platforms such as handheld spectrometers, hand-pushed carts equipped with sensors, tractor-based platforms fitted with multiple cameras and gantry scanalyzer systems that collect spectral information of crops in close range or contact. On the other hand, the remote sensing technique is based on aerial platforms including unmanned aerial vehicles (i.e., drones), manned aircraft and satellites that acquire spectral imagery of crops without making physical contact but at a distance. **Figure 2** was modified from (Pineda et al., 2021).

Non-invasive portable devices, ground-wheeled, motorized gantry scanalyzer systems, agricultural robots and aerial vehicles that deploy a wide range of cameras and sensors, together with high-performance computing are currently required to conduct field phenotyping in a timely and economical manner (**Figure 2**). Together, these platforms are able to phenotype plant characteristics throughout the season in field environments (White et al., 2012; Fritsche-Neto and Borém, 2015; Jimenez-Berni et al., 2018; Furbank et al., 2019; Li et al., 2021).

In recent years, manned and unmanned aerial vehicle (UAV) remote sensing platforms have emerged as convenient high-throughput tools for field phenotyping (Pajares, 2015; Shi et al., 2016; Feng et al., 2021). These remote sensing approaches, particularly UAVs enable quick and non-destructive high throughput phenotyping, with the benefit of adaptable and convenient operation (Yang et al., 2017a). These phenotyping platforms can combine multiple sensors such as digital cameras, infrared thermal imagers, light detection and ranging (LiDAR), multispectral cameras and hyperspectral sensors for various assessments of morphological and physiological plant traits (Gonzalez-Dugo et al., 2015; Yang et al., 2017a; Camino et al., 2018; Roitsch et al., 2019).

Alternatively, field phenotyping can be accomplished on the ground utilizing a fully automated fixed-site phenotyping platform (e.g., Kirchgessner et al., 2017; Virlet et al., 2017; Bai et al., 2019), hand-held sensors, portable spectroradiometers, hand-pushed carts or high-clearance tractors carrying multiple high-resolution sensors to measure phenotypic features non-destructively (Comar et al., 2012; Andrade-Sanchez et al., 2014; Crain et al., 2016). The use of rapid non-invasive portable devices that carry sensors for crop status monitoring has advanced field data collection due to their applicability and ease of operation (Parks et al., 2012; Yang et al., 2014; Condorelli et al., 2018). Recently, field phenotyping has become more flexible by integrating ground-based and aerial platforms (Potgieter et al., 2018; Furbank et al., 2019; Ninomiya, 2022). **Table 1** summarizes the diverse ground-based and aerial field phenotyping platforms, their applications, advantages, and limitations.

**Table 1:** Applications and limitations of field phenotyping platforms.

Phenotyping platform	Examples	Applications	Advantages	Limitations	References
Ground-based platforms					
Fixed-site systems	Field scanalyzers (i.e Rothamsted field scanalyzer, Maricopa field scanalyzer)	Ground cover, canopy height, plant geometry, growth, growth stages, vegetation indices, chlorophyll fluorescence parameters	Unmanned continuous operation with good repeatability, deploy a wide range of sensors, fully automated. Not limited by soil conditions	Expensive, monitor a limited number of plots, limited by weather conditions	Virlet et al., 2017; Burnette et al., 2018
Permanent platforms based on a cable-suspended multi-sensor system	The ETH field phenotyping platform, the University of Nebraska phenotyping system	Monitor canopy cover, canopy height, and traits related to thermal and multi-spectral imaging with selected examples from winter wheat, maize, and soybean	Produce precise, high-resolution images, deploy a wide range of sensors, fully automated	Monitor a limited area of crop, difficult to move, expensive, and limited by weather conditions	Kirchgessner et al., 2017; Bai et al., 2019
Handheld sensors	Point spectroradiometers, thermal sensors, chlorophyll meters, imagers	Estimate chlorophyll fluorescence, canopy temperature, nitrogen status, leaf area, plant height, yield	Ground truth reference to validate aerial measurements (UAVs) and airplanes, low-cost and easy to use	Labour intensive and time-consuming, limited plot coverage, measurement bias	Yang et al., 2014; Andrianto et al., 2017; Garriga et al., 2017
In-field mobile platforms	Phenocart, proximal sensing cart, phenomobiles, manned buggies	Estimate biomass, leaf area index, counting plants, plant height, early vigour, and plant maturity	Manually operated, low-cost, easier to construct, multiple traits evaluations, deploy more sensors, flexibility with payload and view angle geometry; very adaptable	The motorized platforms are costly to construct and run, need technical expertise, hard operation for large-scale experiments. Limited by weather and soil conditions	White and Conley, 2013; Andrade-Sanchez et al., 2014; Deery et al., 2014; Crain et al., 2016
Aerial platforms					
Unmanned aerial vehicles (UAVs)	Broadly classified into Rotocopters, fixed wing systems, parachutes, and blimps	Traits such as canopy cover, canopy height, crop lodging, growth indices, and canopy temperature can be estimated from the imagery	Rotocopters (i.e drones) can deploy a wide range of sensors, including thermal, multispectral, and hyperspectral cameras, high hovering capabilities, better flight time	Lower speeds for image stitching, lens distortion, and overlap of the acquired images can affect orthomosaic, battery use and flying time may be limited by the payload, and operability is limited in windy, wet, dull, variable light, or cold conditions	Sankaran et al., 2015; Zaman-Allah et al., 2015; Chawade et al., 2019; Holman, 2020
Satellite imaging	Digital Globe WorldView-2 satellite, WorldView-3 satellite, RADARSAT-2	In precision agriculture for germplasm evaluation, multi-location yield trials, field observation of crop biophysical parameters, weather predictions	Evaluation of moderate to large-sized trial, multi-location evaluation; provides automated coverage of isolated field trials across a larger geographical area	Affected by weather conditions, resolution, frequency of imaging, takes a long time from image acquisition to access, costly, higher frequency of satellite revisits, cloud cover can interfere with imaging	Tattaris et al., 2016; Yang et al., 2017b; Yang, 2018

Modified from Li et al., 2014 and Deery et al., 2014.

## 4 Traits assessed by sensor platforms and their relevance for field phenotyping

For field phenotyping, traits that have been evaluated by sensors in the field have been reviewed recently by Watt et al. (2020) and include for example; (a) plant morphological development (i.e., including seed establishment and growth of the crop, the timing, and dynamics of flower and fruit development); (b) functional traits that are related to the photosynthetic capacity and carbon uptake during the phenological growth phase; (c) traits related to biotic and abiotic stress resistance/tolerance; (d) traits that determine crop water status (e.g., water uptake and transpiration and water-use efficiency) of plants; (e) yield-related traits and harvest quality of crops (i.e., biomass yield) and (f) the structural and functional root traits (i.e., root architecture). These traits have been previously classified into morphometric and physiological parameters (Qiu et al., 2018). Traits such as plant height, stem diameter, leaf area or leaf area index, leaf angle, stalk length and in-plant space are morphometric parameters. Physiological parameters include traits such as photosynthetic rate, chlorophyll content, water stress, leaf water content, biomass, and salt resistance, which together can impact plant growth. It should be emphasized that different phenotypic traits have specific time frames within the phenological cycle of the plant when they are relevant for the breeder and farmer. Currently, the most researched crops in field phenotyping are economic crops, such as wheat, maize, barley, sorghum, tomato, bean and grape because they have significant economic value for agricultural development. A challenge is to extend phenotyping into the vast range of African crops, some of which may be of only local importance.

Field phenotyping makes use of a variety of sensors due to the large number of phenotypic traits that must be measured. Several conventional and novel sensors such as digital cameras, range cameras, depth cameras, spectral sensors, lidar or laser sensors, thermal sensors, fluorescence sensors, multispectral cameras, hyperspectral cameras and others are employed and integrated for plant trait measurement in field phenotyping (Qiu et al., 2018; Roitsch et al., 2019; Xie and Yang, 2020).

Since plants develop rapidly during their early growth stages, frequent measurements during their establishment are a prerequisite for the quantitative selection of vigour phenotypes. Drones fitted with conventional RGB (red-green-blue) cameras, in combination with advanced image processing pipelines, can automatically detect crop stands (single plants) and determine seed emergence, germination rates and timing under extreme climatic events in the field (Liu et al., 2017).

Most plants display strong morphological changes during their phenological development, which is greatly influenced by the availability of resources and changes in abiotic and biotic factors. Therefore, the development of robust, automated, and precise methods to measure morphological plant traits in field conditions is still required (Gibbs et al., 2017).

The leaf is one of the important components of a plant. It plays a major role in plant growth given that its growing status influences the efficiency of the direct solar energy utilization by plants. Hence, it is a significant parameter in plant phenotyping. Measurements of morphometric parameters of the leaf and other canopy features (i.e., leaf area, stem height, number of tillers, and inflorescence architecture) have been evaluated using non-destructive multi-sensor approaches (Busemeyer et al., 2013; Fiorani and Schurr, 2013; Rahaman et al., 2015). However, the most frequently used geometric measure of plant canopy is the green leaf area index (GLAI), which relates the one-sided green leaf area per unit projected ground area (Chen and Black, 1992). For instance, UAV multispectral imagery has been used to characterize GLAI dynamics of a large maize panel under contrasted environmental conditions and thus holds great potential for yield predictions in breeding programs (Blancon et al., 2019). LAI can also be evaluated, indicating plant coverage, from spectral images (Dammer et al., 2016; Schirrmann et al., 2016).

Plant canopy architecture and other morphological traits of plant organs have been measured concurrently with 3D proximal sensing techniques. A body of recent reviews has compared the performances of the most common 3D sensors for high-throughput plant phenotyping (Li et al., 2014; Qiu et al., 2019). The 3D acquisition devices and approaches commonly used are LiDAR time-of-flight cameras, mono, and multi-view stereo vision and structure-from-motion. The LiDAR sensors can scan and extract morphological traits of plant organs from 3D point clouds. For example, LiDAR was used to estimate plant height, ground cover and above-ground biomass in wheat (Jimenez-Berni et al., 2018). However, LiDAR sensors are expensive (Li et al., 2014), take significant time and there is a need to increase scanning time to increase the spatial resolution. Deploying a UAV-based system may reduce this challenge.

Plant height is a key indicator for canopy structure, yield, carbohydrate storage capacity and lodging occurrence (Holman et al., 2016; Hassan et al., 2019). Additionally, it has significant applications in predicting biomass, identifying plant cultivars, plant stress and phenological stages (Aasen et al., 2015). The traditional method of measuring height using a metre rule is labor-intensive, cumbersome and low throughput. In recent years, the development of drones and imaging sensors that capture high-resolution images has enabled high-throughput plant height estimation. For instance, Holman et al. (2016) estimated wheat height using UAV-based RGB images and terrestrial LiDAR.

Chlorophyll is a vital plant trait because it is strongly related to crop physiological status and may be indicative of photosynthetic rate, crop stress, nutrition status, yield, and plant productivity (Peng et al., 2011; Maimaitijiang et al., 2017). The most popular tools for evaluating vegetation health using visible and near-infrared light are spectral sensors. Chlorophyll meters such as the SPAD-502 are frequently used instruments to measure the relative chlorophyll content. Handheld chlorophyll meters and fluorescence meters have been used to assess plant nitrogen status, photosynthesis, yield and its components in crops (Yang et al., 2014; Andrianto et al., 2017; Fernández-Calleja et al., 2020). Additionally, chlorophyll can be measured using NDVI sensors and portable spectrometers in the field (Bai et al., 2016).

Crop nitrogen content can serve as a proxy for soil fertilizer availability, assisting farmers in precision nitrogen application to the soil. UAV-based hyperspectral imaging and ground-level optical sensors (SPAD-502, Duplex, and Multiplex) have been employed to estimate nitrogen fertilization status in maize (Quemada et al., 2014). In another study, Zaman-Allah et al. (2015) used a UAV equipped with a multispectral sensor (Green, Red, and NIR) to assess low nitrogen stress tolerance in corn. Additionally, vegetation indices (VIs) derived from spectral reflectance data captured by sensors devices such as the CropScan multispectral radiometer (Zhu et al., 2008), handheld spectroradiometers and the FieldSpec (Fitzgerald et al., 2006; Tilling et al., 2007; Feng et al., 2008), Tec5 (Erdle et al., 2013) can accurately measure nitrogen status in wheat and rice.

The above-ground biomass reflects light use efficiency and growth and is vital for carbon stock accumulation and monitoring (Swinfield et al., 2019). Brocks and Bareth (2018) estimated the biomass in barley using RGB images collected by UAV. Thermal infrared sensors are mostly used to detect crop water stress since they can provide temperature information for the crop (Park et al., 2017; Poblete et al., 2018; Bian et al., 2019). Thermal infrared sensors enable the estimation of canopy temperature which is a reflection of plant transpiration and plant water status. Kumar et al. (2020) used a proximal phenotyping cart (phenocart) mounted with low-cost consumer-grade digital cameras to characterize wheat germplasm for drought tolerance under field conditions. Plant yield has been considered an important agronomic trait for field phenotyping. Bascon et al. (2022) estimated rice yield using multispectral images.

The features of the sensors (e.g., spectral resolution, spatial resolution, specificity, and cost) should be considered according to the specific applications, phenotyping needs and context. In the African context, low-cost sensors and analysis pipelines which are not complex would benefit a broader user base for plant phenotypic trait assessments. The most successful trait assessment approach incorporates in time (throughout the crop cycle) and space (at the canopy level) the performance of the crop with respect to capturing resources (e.g., radiation, water and nutrients) and the efficiency of resource utilization (Araus et al., 2008). The aforementioned traits are discussed here with specific examples of sensors and automated measurement approaches used for their evaluation in the field (see **Table 2**). The advantages and limitations of each type of sensor are indicated.

**Table 2:** Emerging high-throughput phenotyping techniques and integrated sensor platforms applicable for plant trait assessment for field phenotyping.

Sensor	Examples	Crop species	Trait/phenotypic parameter	Applications	Advantages	Limitation	References
Hyperspectral sensor	VNIR, SWIR	Rice, Wheat	Nitrogen status, nitrogen use efficiency, water content, yield estimation, canopy components	Evaluate spectral properties, explore hyperspectral bands, estimate indices for fertilizer accumulated in plant organs, early detection of plant stress	Accurate estimation of nitrogen content and other biochemical or physiological status	Update model for new crop species, image processing is challenging, sensors are costly, large data size	Seiffert et al., 2010; Deery et al., 2014; Sadeghi-Tehran et al., 2021; Wang et al., 2021
Thermal sensor	Thermal infrared sensor, near-infrared camera, FLIR sensor	Wheat	Canopy temperature, drought tolerance, water use efficiency	Monitor crop temperature for abiotic stresses e.g., drought tolerance	Low-cost, precise and reliable in repeated experiments	Environmental factors have an impact on performance, very small temperature variations are undetectable, and cameras with higher resolution are heavier	Costa et al., 2013; Deery et al., 2019; Sagan et al., 2019
Visible light sensor	RGB sensor, visible light camera	Rice	Shoot growth, phenology, greenness, plant vigour, leaf area	Visible phenotype parameters, classification of crop organs, greenness, growth and health, time series of vegetation indices	Affordable sensors are available	Visual spectral bands and properties are limited, Changes in illumination conditions cause image blur and noise errors	Kipp et al., 2014; Guo et al., 2015
3D sensor	LIDAR (Light Detection and Ranging) sensor, 3D laser scanner	Maize, Wheat	Plant height, canopy cover, above-ground biomass, crop architecture	Extract morphological traits of plants organs from 3D point clouds; measuring crop height and volume	3D plant information can be quickly captured through close-range observation	LIDAR can be sensitive to small variations in path length, field applications can be challenging	Müller-Linow et al., 2015; Guo et al., 2018; Jimenez-Berni et al., 2018; Qiu et al., 2019
Fluorescence sensor	Fluorescence camera, LIFT fluorometer	Wheat	Photosynthetic capacity, chlorophyll content, quantum yield	Measure photosynthesis, chlorophyll, water stress	Automatic and rapid measurement of photosynthetic parameters	Limited for UAV imagery, can be affected by background noise, difficult to use in the field	Chaerle and Van Der Straeten, 2000; Zendonadi dos Santos et al., 2021
Multispectral sensor		Sorghum, Maize	Disease resistance, nutrient use efficiency, N content, biomass, grain yield	Multiple plant responses to nutrient deficiency, water stress, diseases, etc.,	High-resolution, fast	Sensors can be expensive, limited to a few spectral bands	Zaman-Allah et al., 2015; Zhao et al., 2021
Spectrometer		Maize	Water content, seed composition, yield	Leaf and canopy growth, disease evaluation, leaf area, chlorophyll content, canopy temperature, and crop responses	Handy and easy to use, inexpensive	The quality of the data may be affected by soil background, spectral mixing could occur, and sensor calibration required	Cozzolino, 2014; Andrianto et al., 2017; Chivasa et al., 2020; Cavaco et al., 2022

Modified from Zhao et al., 2019.

## 5 Overview of the status of field phenotyping in Africa

Despite the recent advances in high-throughput field phenotyping based on the non-destructive analysis of plant traits, Africa has yet to consolidate the gains of these cutting-edge technologies for research into agricultural productivity. In terms of the deployment of high-end field phenotyping tools and approaches, Africa cannot keep pace with many regions, even in the era of artificial intelligence (AI), ‘internet-of-things’ (IoT) and technological advancements, although more affordable and lean phenotyping systems are now becoming available. Community-wide surveys and exchanges conducted by the International Plant Phenotyping Network (IPPN) and European Infrastructure for Multi-Scale Plant Phenomics and Simulation (EMPHASIS) within the growing phenotyping community in recent years have identified focus areas to assess the status of global plant phenotyping and crucial bottlenecks in the emerging field.

The major bottlenecks for developing field phenotyping in Africa were non-invasive phenotyping approaches, data management and cost among others (IPPN, 2016; Rosenqvist et al., 2019). This survey further reveals that in terms of using high-intensity field approaches (e.g., automation, robotics, image analysis and data storage management) for field phenotyping, Africa ranks lowest around the world. A recent survey conducted in the framework of the IPPN and EMPHASIS projects in 2020 (IPPN, 2020) which is reported by Yang et al. (2020) and Fahrner et al. (2021), indicated that Africa is still behind in the implementation of high-throughput field phenotyping. This highlights the need for a broader deployment of high-throughput field phenotyping techniques, which are essential enablers or resources for agricultural sciences and breeding to address upcoming crop production challenges.

The IPPN over the years has been promoting the idea of strengthening modern plant phenotyping in African countries by giving travel grants to Africa and inviting students and researchers for International Plant Phenotyping symposia and internships. However, only a few institutional members are identified for collaboration in the region. In recent times, there have been some high-throughput field phenotyping research and initiatives in African countries such as South Africa, Ghana, Senegal, Morocco, Nigeria, Ethiopia, Kenya, Egypt, and Zimbabwe which is encouraging for the emerging field and will be highlighted in this review (see section 5.2 and **Table 3**).

**Table 3:** Summary of some major characteristics of field phenotyping activities implemented in some African countries.

Region	Country	Area of high-throughput field phenotyping research	Prospects	Reference/web link
**West Africa**	Ghana	Exploration of digital agriculture, deployment of low-cost sensors and technologies for breeding, exploration of remote sensing for precision agriculture, GIS	Digital agriculture, low-cost precision agriculture, and breeding, use of high-throughput tools	Hall et al., 2018; Kpienbaareh et al., 2019; Kassim et al., 2022; Sie et al., 2022; https://ftfpeanutlab.caes.uga.edu/Research/variety-development/high-throughput-phenotyping-in-senegal–ghana-and-uganda.html
	Senegal	Exploration of digital agriculture, exploration of UAV imagery, multi-spectral imaging, GIS	Development of high-throughput approaches, digital agriculture, low-cost precision breeding	Dingkuhn et al., 2015; Gano et al., 2021; https://www.devdiscourse.com/article/other/523595-senegals-embrace-of-the-digital-revolution-in-agriculture-marks-the-way-forward-for-africa
	Nigeria	Use of field mobile agricultural robots, digital imaging, remote sensing, machine learning, GIS, site-specific analytics, drone imagery	Development of high-throughput approaches, digital agriculture, low-cost precision breeding, deployment of digital technologies and innovations	Ifeanyieze et al., 2014; Daniel et al., 2016; Ejikeme et al., 2017; Iseki and Matsumoto, 2019; Alabi et al., 2022; Izuogu et al., 2023; https://nitda.gov.ng/wp-content/uploads/2020/11/Digital-Agriculture-Strategy-NDAS-In-Review_Clean.pdf
**North Africa**	Morocco	High-throughput phenotyping, precision field-based phenotyping platform for drought/heat tolerance, development of quinoa phenotyping methodologies, expanding the precision and prediction value of phenotyping/genotypic data for new germplasm emerging from the wheat, adding an HTPP system for wheat abiotic stresses	Expanding phenotyping capabilities, low-cost precision breeding, deployment of digital technologies and innovations, expansion in remote sensing capabilities	Bijaber et al., 2018; Danzi et al., 2019; Bouras et al., 2020; Jabir and Falih, 2020; Laachrate et al., 2020; Quahir et al., 2022; https://www.fao.org/in-action/plant-breeding/nuestrosasociados/africa/morocco/es/; https://www.icarda.org/research/projects/precision-field-based-phenotyping-platform-droughtheat-tolerance-morocco-pwpp
	Egypt	High-throughput precision phenotyping for improvement of drought and salt tolerance in wheat genotypes, implementation of digital technology (mobile applications) for field phenotyping, AI-enabled system to enhance agriculture process, satellite imagery for crop monitoring	Expanding phenotyping capacities, low-cost precision agriculture, and breeding	El-Shirbeny et al., 2014; Elsayed et al., 2017; Shokr, 2020; Bahn et al., 2021; Elmetwalli et al., 2022; Elsafty and Atallah, 2022; Abdelnabby and Khalil, 2023; Mahdy and Ahmad, 2023; Sayed et al., 2023; https://globalrust.org/geographic/egypt; https://www.fao.org/e-agriculture/news/egypt-turns-fao-digital-transformation-agriculture; https://dailynewsegypt.com/2021/12/07/government-launches-ai-enabled-system-to-enhance-agriculture-process/
**Southern Africa**	South Africa	Deployment of field scanalyzer (FieldScan) for spectral crop measurement, remote sensing for precision agriculture	Expanding phenotyping capacities, low-cost precision agriculture, and breeding, advancing remote sensing capabilities	Mutanga et al., 2016; Brewer et al., 2022a, b; Buthelezi et al., 2023
	Zimbabwe	UAV-based high-throughput phenotyping, multispectral remote sensing in maize varietal response to maize streak virus (MSV) disease, high-throughput phenotyping of maize performance under phosphorus fertilization, remote sensing methodologies for crop monitoring under conservation agriculture	Expanding phenotyping capacities, low-cost precision agriculture, and breeding	Kefauver et al., 2015; Zaman-Allah et al., 2015; Gracia-Romero et al., 2018; Musungwini, 2018; Buchaillot et al., 2019; Chivasa et al., 2020; Gracia-Romero et al., 2020; Shonhe and Scoones, 2022; Parwada and Marufu, 2023;
**East Africa**	Kenya	Satellite-based assessment of maize yield variations in smallholder farms, GIS and remote sensing capabilities	Expanding phenotyping capacities, low-cost precision agriculture, and breeding	Kefauver et al., 2015; Kotikot and Onywere, 2015; Zaman-Allah et al., 2015; Burke and Lobell, 2017; Manzi and Gweyi-Onyango, 2021
	Ethiopia	GIS for precision agriculture, imaging technologies for crop trait analysis	Expanding phenotyping capacities, low-cost precision agriculture, and breeding	Alemaw and Agegnehu, 2019; Bontpart et al., 2020; Debesa et al., 2020; Beyene et al., 2022; Debalke and Abebe, 2022

Like in many developing countries, field phenotyping in African countries is mostly based on conventional and traditional methodologies which rely heavily on manually recorded measurements of phenotypic data or visual assessment of plant parameters. It entails manually inspecting crops and measuring several crop characteristics that affect yield traits, including plant height, number of tillers, leaf color, leaf shape, leaf area index (LAI), chlorophyll content, growth stages, above-ground biomass and stress tolerance (Gedil and Menkir, 2019; Bongomin et al., 2022; Badu-Apraku et al., 2023). In practice, in traditional field phenotyping, breeders or research evaluators inspect the trial fields and rate the plots according to how they feel, taste, smell, and appear (Kim, 2020). Such phenotyping methods have several disadvantages such as being low-throughput, time-consuming, laborious, expensive and error-prone (Chapu et al., 2022; Xiao et al., 2022). Although these methods have been beneficial in developing new crop cultivars and improved yields, it is crucial that more effective phenotyping methods be used to increase the accuracy of data collection.

In parallel, field phenotyping is undertaken to evaluate the agronomic performance of crops in breeding programs, germplasm collections and in biotechnology programs to deliver improved cultivars that can cope with environmental stresses (e.g., Asare-Bediako et al., 2019; Gedil and Menkir, 2019; Rezende et al., 2020; Kavhiza et al., 2022). These phenotyping research targets are focused on key crops for food security but are predominantly low-throughput phenotyping based on field trials. In sub-Saharan Africa, breeding programs championed by the Alliance for a Green Revolution in Africa (AGRA) have been dedicated to priority crops such as rice, maize, cassava, yam, beans, cowpea and vegetables under various regional breeding networks for improved varieties and seed systems (FAO, 2011; AGRA, 2019).

Previous studies have used a variety of calibration data, including ground-based survey methods and crop model simulations, to predict yield in smallholder systems (Burke and Lobell, 2017; Ogutu et al., 2018). However, there has been emerging evidence in SSA suggesting inaccurate farmer-reported crop production estimates in smallholder production systems (World Bank, 2010; Gourlay et al., 2017; Abay et al., 2019; Wahab, 2020). These anomalies in crop yield estimation at smallholder, country and regional levels can cause price fluctuations (i.e., inflation), wrong national policy decisions and food insecurity among others. High-throughput and/or digital phenotyping might offer a better estimation of regional and national crop production.

Recent advances in sensor technology and the availability of free high-resolution (spatial and temporal) multispectral satellite images have also presented an opportunity to predict the yield of maize (Chivasa et al., 2017) and detect leaf spot diseases in groundnut (Sie et al., 2022), adaptation responses to early drought stress in sorghum (Gano et al., 2021) as well as mapping spatial distribution on a near real-time basis for a region, which hitherto was not feasible.

### 5.1 Field phenotyping initiatives and programs in Africa

Despite the low implementation of high-throughput field phenotyping in Africa, there are some efforts by research organizations to adopt the technology in some countries. Prominent among these initiatives is a global network for precision field-based wheat phenotyping. (https://globalrust.org/content/global-network-precision-field-based-wheat-phenotyping). Based on a global network of wheat partners, field phenotyping platforms are being developed with the support of the CGIAR research program on wheat and co-investing national agricultural research centers around the world, including some African countries such as Kenya, Ghana, Nigeria, Ethiopia, and Morocco.

The main goal of this network is to generate high-quality phenotypic data to assist plant breeders in developing disease and drought-resistant, high-yielding wheat varieties with a broad genetic base and maximizing the potential of new genotyping technologies. Additional but vital goals are to share knowledge and germplasm to accelerate new germplasm development and dissemination as well as develop capacities of breeders and plant scientists in precision field phenotyping. Some examples of these field phenotyping interventions being implemented include the development and application of precise phenotyping approaches, standardized protocols and novel tools for heat stress assessment in Sudan, *Septoria tritici* blotch in durum wheat in Tunisia (Ben M’Barek et al., 2022), *Septoria tritici* blotch in durum wheat and wheat rusts in Ethiopia (Kidane et al., 2017; https://globalrust.org/content/sources-resistance-septoria-tritici-blotch-identified-ethiopian-durum-wheat), heat and drought tolerance in spring wheat in Morocco, yield potential in Egypt and Zimbabwe and drought and yield potential in Kenya (https://globalrust.org/content/global-network-precision-field-based-wheat-phenotyping).

Additionally, low-cost high-throughput phenotyping tools for field selection for disease, drought and crop variety performance are currently being developed. These tools will be used in breeding programs in Senegal, Ghana and Uganda and will serve as “centers of excellence for peanut breeding” in West and Eastern Africa (https://ftfpeanutlab.caes.uga.edu/Research/variety-development/high-throughput-phenotyping-in-senegal–ghana-and-uganda.html).

In West Africa, the field phenotyping network, since its inception in 2016 in the sub-region, has implemented high-throughput UAV (drone-based) phenotyping methodologies which are functional for sorghum, cowpea, pea nut and pearl millet (Gano et al., 2021; Audebert et al., 2022). The network is advancing breeding activities through ‘fine phenotyping’, varietal evaluations in diverse environments to identify hot spots for specific stresses, including farmers’ fields to test promising breeding lines in participating countries such as Senegal, Ghana, Mali and Burkina Faso.

The establishment of the network has facilitated infrastructure development, equipment acquisition, data management paired with long-term training of dedicated students, technicians and breeders capable of doing both breeding and carrying out high-throughput phenotyping measurements. In the subregion, three sites have been chosen as prospective hubs for high throughput phenotyping. Each hub including Bambey (ISRA research station, Senegal), Sotouba (IER research station, Bamako, Mali) and Farako-ba (INERA research Station, Bobo Dioulasso, Burkina Faso) exemplifies the diversity of soil and climate conditions in the region. According to Audebert et al. (2022), the network setup in Senegal is the most advanced while Mali and Burkina Faso lag behind mainly due to limited phenotyping equipment and funding challenges.

Similarly, the Regional Study Centre for the Improvement of Drought Adaptation (CERAAS) in complementing the field phenotyping initiatives of the West African field phenotyping network, has developed robust UAV imagery-based data collection and spatial modelling methodologies to accurately measure key traits of cereal crops to advance plant breeding programs. UAVs equipped with a multispectral imaging system coupled with a fully automated image processing pipeline can indirectly measure agronomic and phenological characteristics of cereal crops in agricultural field trials (Mbaye et al., 2022).

Moreover, to advance the promotion and advancement of precision agriculture (PA) in Africa, the African Association for Precision Agriculture (AAPA), an initiative of the African Plant Nutrition Institute (APNI) is spearheading this goal (https://paafrica.org/AAPA). Since its establishment in 2020, the AAPA has worked in partnership with academia, research institutions, agri-food industry, financial institutions, and public and private sector organizations to develop and scale up PA strategies and innovations through sustainable integration into African agriculture to address food security (i.e., reduce yield gaps) climate change, and land degradation challenges.

### 5.2 Field phenotyping research in African countries

#### 5.2.1 The case in Ghana

Digitalization of Agriculture is a new trend facilitated by digital platforms aimed at transforming small scale agriculture by providing agricultural services to smallholder farmers in Ghana (Atanga, 2020; Abdulai et al., 2023). These digital platforms include simple devices such as mobile phones or radio to a more sophisticated devices (e.g., field sensors, GIS, drones, field sensors, machinery sensors and diagnostics precision systems).

In Ghana some of the notable digital platforms transforming the small-scale farming sector include the TROTRO Tractor Limited (an agritech company) that combines mechanization with IoT and technology to make agricultural machinery (i.e., tractors and combined harvesters) available, accessible, and affordable to farmers thereby enhancing their efficiency and productivity (https://www.trotrotractor.com). The use of remote sensing as a decision support system (DSS) tool to optimize irrigation and farm management towards increasing yields has also been demonstrated (Kpienbaareh et al., 2019). These innovations primarily address the numerous issues smallholders and rural farmers confront in the present food systems, such as climate change, low access to inputs and restricted access to information (Degila et al., 2023).

As in many African countries breeding and field phenotyping is mostly based on conventional manual methods. However, to evaluate crop performance and improve breeding competitiveness, modern technologies using high-throughput techniques are being implemented but at a slow pace (e.g., Hall et al., 2018; Kassim et al., 2022; Sie et al., 2022). For instance, the responses of two populations of groundnut genotypes with various maturities to early and late leaf spot diseases were assessed under field conditions using UAV imagery (Kassim et al., 2022). In another breeding program, a smartphone-based RGB images detected leaf spot resistance and predicted yield in groundnut (Sie et al., 2022). In a resource constraint economy, Ghana is faced with numerous challenges such as lack of research funding, phenotyping infrastructures and technical personnel among others that can advance rapid characterization of agriculturally relevant traits (e.g., growth, yield, stress resistance). To increase its phenotyping capabilities will require a concerted effort from all stakeholders across the crop production value chain.

#### 5.2.2 The case in Senegal

Senegal is making strides in precision agriculture by employing digital tools to address crop production challenges (https://www.apni.net/wp-content/uploads/2020/02/WAFPA-Tine.pdf). Even though advancement in modern breeding and field phenotyping methodologies has been slower and predominantly based on conventional methods (e.g., Dingkuhn et al., 2015), the use of drones for agricultural monitoring (i.e., stress detection, disease surveillance, crop performance) aided by high-throughput phenotyping has been exploited thanks to initiatives by the CERAAS and West African field phenotyping network. For instance, UAV multi-spectral imaging has been employed for the estimation of shoot biomass, leaf area index (LAI) and plant height of West African sorghum varieties under severe drought conditions (Gano et al., 2021). The drone-based field phenotyping approach developed could help identify essential traits and cultivars for drought tolerance in sorghum breeding. The main challenges confronting crop field phenotyping in Senegal are lack of equipment, technical personnel and funding (Audebert et al., 2022). However, Senegal being a hub for field phenotyping in West Africa, has the potential to increase its field phenotyping capabilities in the future.

#### 5.2.3 The case in Nigeria

According to a recent review by Izuogu et al. (2023), the digitalization of agriculture in Nigeria has reduced middlemen’s participation in agriculture, offered small-holder farmers opportunities to improve their productivity and markets, and strengthened the connections between extension and research facilities. The authors demonstrated that for effective digitalization of agriculture, training was required in the areas of skills development, use of demand-driven digital services, digital privacy, and security issues. The challenges of digitalization of agriculture identified were lack of technical expertise, inadequate infrastructure, and high purchase and maintenance costs. The use of remote sensing techniques for precision crop production and monitoring has been implemented but to a lesser extent. Ifeanyieze et al. (2014) have previously reviewed the remote sensing techniques needed for the smooth implementation of precision crop management by farmers as a climate change adaptation strategy in Nigeria. Few research groups have utilized remote sensing techniques for field phenotyping. For instance, Ejikeme et al. (2017) used a satellite-based crop prediction model to estimate crop statistics of major crops including rice, cassava, yam, and maize. Recently, the Institute of Tropical Agriculture (IITA) through its collaborative soybean breeding programs has implemented machine learning (ML) models and multispectral high-resolution UAV imagery to aid rapid high-throughput phenotypic workflow for soybean yield estimation (Alabi et al., 2022). Other breeding programs used manual field evaluation coupled with digital imaging analysis for phenotyping tomato breeding population (Daniel et al., 2016).

The use of a handheld optical NDVI sensor for the evaluation of shoot biomass in field-grown staking yam has been implemented (Iseki and Matsumoto, 2019). Altogether, Nigeria has great potential for improving its field phenotyping capabilities.

#### 5.2.4 The case in Morocco

**Figure 3:**
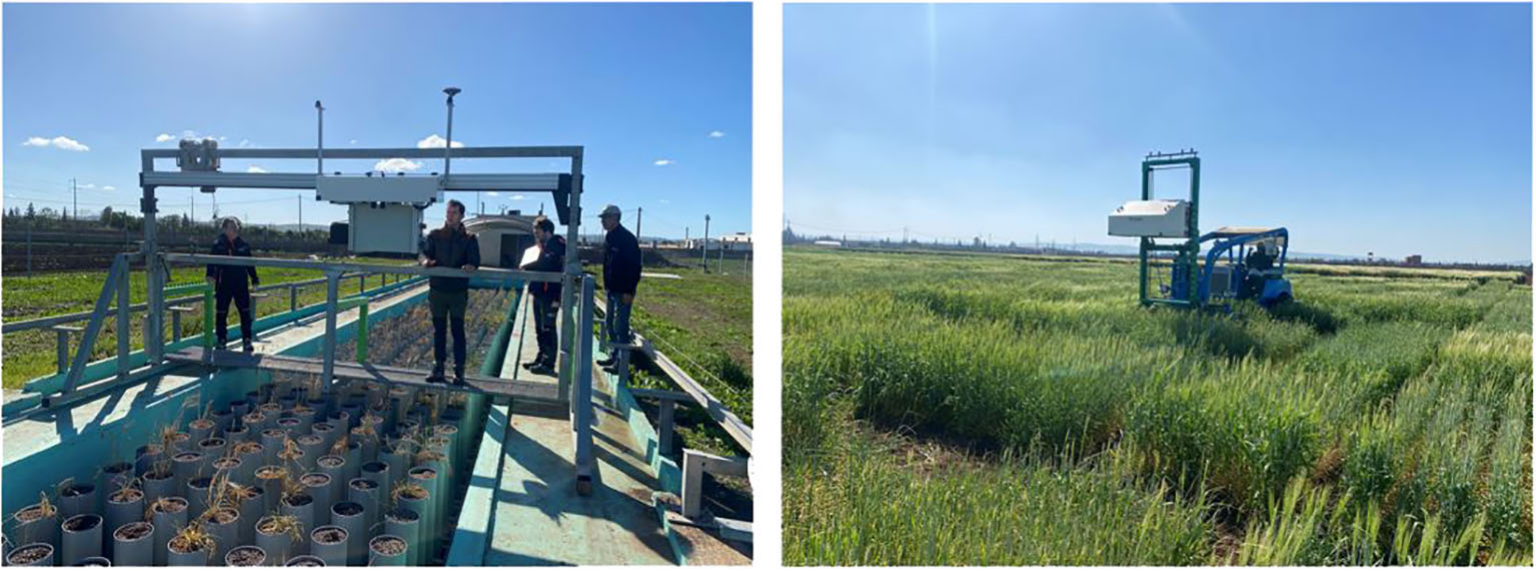
The ICARDA's precision field phenotyping platforms installed at Sidi el Aidi (Settat) in Morocco. Images are in courtesy of Andrea Visioni of ICARDA-Morocco.

Morocco is among the few African countries well-positioned for widespread agricultural digitalization for precision agriculture and field phenotyping to increase crop production and cope with adverse environmental conditions such as drought. Jabir and Falih (2020), recently reviewed the state of digital agriculture in Morocco and highlighted the opportunities and challenges that need to be addressed. The design and implementation of a wireless sensor network (WSN) and decision support tools (i.e., drones) for monitoring the agricultural environment have been demonstrated (Jabir and Falih, 2020). Nevertheless, challenges such as sensor deployment and inadequate software analytics still exist (Kobo et al., 2017). Morocco is home to the International Centre for Agricultural Research in the Dry Areas (ICARDA’s) phenotyping facilities (ICARDA phenotyping platforms in Morocco), including a precision phenotyping platform at Sidi el Aidi (Settat) (**Figure 3**) and a phenomobile system (PhenoBuggy) situated at the main research station in Marchouch (Rabat) designed for drought and heat stress tolerance studies (https://www.cgiar.org/news-events/news/icardas-phenotyping-facilities-a-game-changing-solution-for-abiotic-stress-tolerance-in-crops/). The PhenoMA is another high-throughput phenotyping platform currently installed in Benguerir (Quahir et al., 2022). Field phenotyping using various remote sensing techniques has been deployed for drought monitoring (Bijaber et al., 2018; Bouras et al., 2020; Laachrate et al., 2020), and grain yield prediction (Belmahi et al., 2023).

#### 5.2.5 The case in Egypt

Digital agriculture appears promising in addressing the major challenges facing the agri-food sector in Egypt and across the Middle East and North Africa (MENA) countries (Bahn et al., 2021). Available evidence indicates that the adoption of digital and precision agriculture technologies is still in its infancy and is typically driven by high-value agricultural production (Elsafty and Atallah, 2022; Sayed et al., 2023). However, Egypt has made strides in the utilization of modern technologies for agricultural crop management employing big data in tandem with cloud support systems, IoT, UAVs, satellite imagery, AI, machine learning, and remote sensing (Shokr, 2020; Abdelnabby and Khalil, 2023; Sayed et al., 2023). Typical high-throughput field phenotyping methodologies has been implemented in various crops for quantifying wheat characteristics in the Nile Delta (Elmetwalli et al., 2022) and estimating the growth performance and yield of soybean exposed to different drip irrigation regimes under arid conditions (Elmetwalli et al., 2020). Additionally, remote sensing techniques based on thermal imaging and passive reflectance have been used to estimate the crop water status and grain yield in wheat (El-Shirbeny et al., 2014; Elsayed et al., 2017).

#### 5.2.6 The case in South Africa

The agricultural sector in South Africa has been developing and moving towards becoming a knowledge-intensive enterprise due to new innovations and technologies incorporated in the digital economy (Baumüller and Kah, 2019; Born et al., 2021; Smidt and Jokonya, 2022). Due to this transformation, conventional production methods have gradually been replaced with more advanced, efficient and innovative systems (e.g., remote sensing) for crop breeding and phenotyping (Mutanga et al., 2016).

Field phenotyping using modern high-throughput infrastructures and precision agriculture techniques is better developed in South Africa compared to other countries on the continent (Nyaga et al., 2021; Mukhawana et al., 2023). Some research groups are making efforts in championing field phenotyping and precision agriculture through workshops and implementation of UAV remote sensing applications and other approaches for agricultural monitoring (stress detection, nutrient and irrigation management) (https://www.fabinet.up.ac.za/index.php/research-groups/remote-sensing). For example, the Forestry and Agricultural Biotechnology Institute (FABI) and the Agricultural Research Council (ARC) (https://www.arc.agric.za/Pages/Home.aspx) are committed to building phenotyping infrastructures and disseminating emerging technologies for agricultural development.

Various remote sensing applications have been employed targeted at different scales of crop monitoring (e.g., crop water use efficiency) in precision agriculture (e.g., Munghemezulu et al., 2023; Wellington, 2023). For instance, foliar temperature and stomatal conductance have been used as indicators of water stress in maize based on optical and thermal imagery acquired using a UAV platform (Brewer et al., 2022a). The utility of multispectral UAV imagery as proxy for predicting chlorophyll content of maize at various growth stages in smallholder farming systems has been reported (Brewer et al., 2022b). The physiological processes of the maize canopy are intimately tied to and influenced by LAI, which is closely related to its productivity (Peng et al., 2021). Another study has focused on estimating the LAI of maize in smallholder farms across the growing season using UAV-derived multi-spectral data (Buthelezi et al., 2023). Maize is a major crop in South Africa, therefore, significant research on the crop using high-throughput techniques will aid in developing improved cultivars for farmers. South Africa has a great potential for becoming the field phenotyping hub of Africa due to the massive investment in modern technologies.

#### 5.2.7 The case in Zimbabwe

In Zimbabwe, the implementation of digitalized agriculture is low and tilted toward commercial farmers than smallholder community farmers (Parwada and Marufu, 2023). Specifically, highly literate, and resource-rich farming communities tend to use digitalized agriculture more frequently than farmers with lesser resources. At the communal level, farmers use mobile phones to obtain farming information relating to crop management, climate, and weather information (Musungwini, 2018; Zimbabwe Centre For High Performance Computing, 2021). The application of modern digital agriculture tools and infrastructure (i.e., sensors, robotics, AI, UAVs, and other advanced machinery is common in a few well-resourced commercial farms notably, those managed by large multinational companies (Shonhe and Scoones, 2022). Parwada and Marufu (2023) recently reviewed the challenges and opportunities for digitalization of the Zimbabwean agriculture. Key challenges such as lack of high-throughput infrastructures, digital illiteracy, and strict regulations for drone deployment among others have been highlighted for limiting digital agriculture applications. However, according to the authors, Zimbabwe have the potential for improving its digital agriculture for crop management, yield prediction, disease detection, climate forecasting, and soil management through precision agriculture. In recent years, few high-throughput phenotyping has been implemented in Zimbabwe using RGB picture vegetation indexes (Kefauver et al., 2015), and multi-spectral imaging for field phenotyping of maize (Zaman-Allah et al., 2015). Other studies include remote sensing methodologies for crop monitoring under conservation agriculture (Gracia-Romero et al., 2018; Gracia-Romero et al., 2020), affordable UAV-based RGB phenotyping techniques for evaluating maize performance under low nitrogen conditions (Buchaillot et al., 2019), and accelerating crop improvement in response to changing climate conditions employing UAV-based multispectral phenotyping for disease resistance in maize (Chivasa et al., 2020). Zimbabwe is among the few African countries capable of advancing its field phenotyping capabilities in the future.

#### 5.2.8 The case in Kenya

Although there are several technologies currently available to Kenya’s agricultural sector they have not yet become widely used (Osiemo et al., 2021). Large-scale adoption of digital solutions is hampered by a lack of digital literacy and infrastructure. Only a few research groups are skilled in using and maintaining back-end service operations like data management, blockchain, machine learning, IoT, GIS, and drones (Osiemo et al., 2021). However, the application of GIS and remote sensing techniques have been used to map frost hotspots for mitigating agricultural losses (Kotikot and Onywere, 2015), climate-smart crop management (Manzi and Gweyi-Onyango, 2021), and assessment of yield variations and its determinants in smallholder systems (Burke and Lobell, 2017). Similarly, high-throughput phenotyping platforms based on multi-spectral imaging and RGB vegetation indices have been implemented for field phenotyping of maize (Kefauver et al., 2015; Zaman-Allah et al., 2015). Kenya has the potential of expanding its phenotyping capacities through low-cost precision agriculture and breeding.

#### 5.2.9 The case in Ethiopia

Digital agricultural innovations in precision agriculture have the potential to increase productivity while minimizing harmful environmental impacts along the value chains of agriculture and the food systems in Ethiopia (Alemaw and Agegnehu, 2019; Tamene and Ashenafi, 2022). In recent years, there have been some improvement in digital infrastructure in Ethiopia (Abdulai, 2022). However, the majority of Ethiopia’s smallholder farmers have limited access to digital farming technologies (Tamene and Ashenafi, 2022). According to Tamene and Ashenafi (2022), several challenges such as inadequate technological capacity, limited funding to develop and disseminate digital tools and lack of data sharing channels hampers the development of digital agriculture in Ethiopia. These barriers restrict the deployment of modern technologies for crop breeding and field phenotyping. Field phenotyping has relied largely on conventional methods as in the studies of eco-geographic adaptation and phenotypic diversity of Ethiopian teff across its cultivation range (Woldeyohannes et al., 2020) and genetic diversity in Ethiopian Durum Wheat (Mengistu et al., 2018). Field phenotyping using high-throughput techniques has been introduced in recent times. Remote sensing and GIS based methods has been used as crop yield predictors in wheat and maize (Beyene et al., 2022; Debalke and Abebe, 2022) as well as physical land suitability analysis for major cereal crops (Debesa et al., 2020). In essence, Ethiopia has the potential to accelerate its phenotyping capabilities. **Table 3** summarizes some key field phenotyping activities that exist in the African countries discussed in this review.

### 5.3 Current developing field phenotyping platforms in Africa

UAVs have been selected as the technical solution that is most suited for deployment across sites and trials throughout the several initiatives that made it possible for the West African field phenotyping network to get started (Audebert et al., 2022). For instance, in Senegal, the UAV platform comprises a FeHexaCopterV2 hexaCopter UAV system (Flying Eye Ltd., Sophia Antipolis, France) fitted with three cameras mounted on a two-axis gimbal pointing vertically downward. The camera consists of an RGB ILCE-6000 digital camera (Sony Corporation, New York, NY, USA), AIRPHEN multispectral camera (Hiphen, Avignon, France), and infrared thermographic camera Tau 2 (Flir system, Oregon, USA) that collects spectral imagery of crops such as sorghum, pearl millet and peanut and cowpea (Gano et al., 2021).

The Agricultural Research Council (ARC) of South Africa has installed a Phenospex planteye multispectral 3D laser scanner (i.e., the first of its kind in Africa) in the field (https://phenospex.com/products/plant-phenotyping/fieldscan-high-throughput-field-phenotyping/fieldscan-3d-spectral-plant-measurements-in-the-field-south-africa/). This state-of-the-art facility is fully automated, carrying a high-resolution sensor that combines the strength of 3D vision with the power of multispectral imaging. It captures plant data non-destructively and delivers precise and accurate plant parameters in real-time. Plant phenotypic features such as digital biomass, plant height, 3D leaf area, projected leaf area, leaf area index, leaf inclination, etc., can be measured. The spectral information allows for the quantification of plant health, disease, senescence, N-content, chlorophyll levels, etc. Therefore, this phenotyping facility could assist in the characterization and development of varieties with improved biotic and abiotic stress resistance for key crops such as grapefruit, sunflower, green maize and other cereals in Southern Africa.

Recently, a unique close-to-field high-throughput plant phenotyping platform “PhenoMA’’ has been installed in Benguerir, in the arid region of Morocco by the Mohammed VI Polytechnic University. PhenoMA consists of a 1440 fully automated lysimetric mini-plots system that can track the dynamics of water use and simulate drought scenarios. A critical component is a fully autonomous phenotyping robot (Hiphen PhenoMobile) that enables plant measurements at the canopy scale, using a range of sensors including RGB, multispectral, infrared (IR), and LiDAR cameras to monitor canopy development (Quahir et al., 2022).

Overall, due to the rich agricultural biodiversity of Africa, phenotyping in Africa has great potential to contribute to the development of improved crop varieties and enhanced food security. The utilization of high-throughput tools can boost the elucidation of new agriculturally proven traits and catalogue these phenotypes in their natural environment.

## 6 Challenges limiting the application of high-throughput field phenotyping in Africa and the way forward

The application of emerging field phenotyping technologies has the potential to accelerate plant breeding efforts and crop production in Africa. On the other hand, most of these approaches reviewed here are at best relatively new or unknown to some of the plant science community in Africa. Field phenotyping is a critical component of crop improvement but remains a major bottleneck in African agriculture, as is the case globally. Some of the key challenges limiting the application of high-throughput field phenotyping in Africa are highlighted below.

### 6.1 Lack of appropriate high-throughput field phenotyping approaches

Phenotypic analysis has become a major limiting factor in genetic and physiological analyses in plant sciences as well as in plant breeding in Africa. The inadequate phenotyping infrastructures and software analytical tools that can be used by agricultural practitioners to make sense of simple to complicated phenotypic datasets have contributed to the low implementation of high-throughput phenotyping. The operational complexity to support both data acquisition and analysis has limited the use of these platforms for research activities worldwide (Chapman et al., 2014), including developing continents like Africa. To this end, training in image analytics, software, and computer vision to provide a new generation of skilled personnel must be implemented by African governments, universities, and the private sector. Phenotyping advancement is critical for current breeding progress for crop improvement in Africa. While the development of efficient high-throughput field phenotyping remains a challenge for future breeding progress, the growing interest in low-cost solutions for remote-sensing approaches, machine vision, as well as data management, may facilitate technological adoption.

### 6.2 Cost of phenotyping infrastructures and maintenance

As a developing continent comprising highly indebted poor countries (HIPC) (Henri, 2019) and faced with multi-faceted economic hardships, the major limitation to the adoption and implementation of high-tech field phenotyping in Africa is the perceived high entry costs associated with the longer-term footprint of prototypical platforms (Reynolds et al., 2019). In several African countries, especially those discussed in this review, basic phenotyping tools and infrastructure even for the simplest field measurements and experimentation are scarce.

This prevents many research organizations in Africa such as IITA, CIAT, and Africa Rice, from implementing demand-driven approaches due to a lack of investment budget or avoiding the significant follow-up costs on maintenance of large phenotyping infrastructures. For instance, the use of ground vehicles, aerial vehicles and gantries may require huge investment costs (Pauli et al., 2016; Vergara-Díaz et al., 2016).

Therefore, the requirements for such specialized equipment may be a bottleneck for widespread use in breeding programs in poor countries. To alleviate this challenge, low-cost concepts and methods of high-throughput phenotyping platforms (HTPPs) (e.g., sensors and platforms) that rely on easy-to-use technology must be disseminated in Africa by identifying demands, and relevance, and adopting the required approach given the current financial constraints. For instance, conventional digital cameras (i.e., digital photography) could provide a more convenient method since they are more affordable, portable, and easy to use (Casadesús and Villegas, 2014).

### 6.3 Limited investment and funding

Limited investments in science, technology, and innovation (STI) on the part of African governments, research institutions (e.g., academia) and the private sector have partly contributed to the poor implementation of high-throughput field phenotyping. The budgetary allocations dedicated to research, development, and innovation are small. For example, in Ghana, a minimum of 1% of gross domestic product (GDP) is applied towards research and development (https://mesti.gov.gh/government-increase-research-funding/). Similarly, in Morocco, the percentage of GDP to research as of 2010 was 0.63% (Hamidi and Benabdeljalil, 2013). This research funding gap is pervasive across the African continent.

Whereas research institutions and universities in developed economies, such as Europe (see https://eppn2020.plant-phenotyping.eu/EPPN2020_installations#/), Australia, North America and Asia, have in recent years invested heavily in large-scale research infrastructure for automated and high-throughput field phenotyping, the same cannot be said for Africa. These large investments for plant phenotyping include funding, research hours and high-throughput installations (Costa et al., 2019; Rosenqvist et al., 2019; https://eppn2020.plant-phenotyping.eu/).

Furthermore, crops grown in Africa are frequently too local to attract international research funding for field phenotyping. Only a few essential African crop commodities, such as cassava and sweet potatoes, are funded solely by extrabudgetary sources. Most of the main staple crops are exclusively funded for phenotyping exploitation outside of Africa.

In addition to the above considerations, African governments and the Science Granting Councils Initiative (SGCI) in sub-Saharan African countries mandated to support the Science Granting Councils (SGCs), must dedicate enough funding for low-cost plant phenotyping research infrastructure in the sub-region in the short to medium term. This could be achieved by developing financing mechanisms and collaborating with private sector partners. Donor support to Africa for agriculture and food security research should also consider projects in modern plant phenotyping and digital agriculture.

### 6.4 Lack of Skilled technical personnel

A serious deficit of skilled technical personnel in the plant sciences and phenotyping ecosystem is evident in African countries. The building up of such competencies and the development of human resource capacity is necessary to operate simple-to-sophisticated equipment to accelerate breeding efforts through high-throughput phenotyping techniques. Another major barrier is the loss of talented and skilled personnel who were trained in developed nations and have contributed to the brain drain due to inadequate job prospects in Africa. Mostly, funds to pay salaries and absorb project operating costs are either limited or insufficient, resulting in a reduction of skilled personnel. Furthermore, due to the inadequacies in research and infrastructure in many African nations, training acquired overseas is sometimes unsuited to local demands. To address this constraint, digital agricultural competencies and sensor technologies should be integrated into undergraduate and postgraduate learning curricula to allow students to specialize in digital agriculture through their projects. This will create a plethora of career opportunities for competent skilled personnel who can adapt to the emerging technologies for field phenotyping.

### 6.5 Regulations controlling emerging technologies

Emerging technologies such as UAVs offer the advantages of being flexible, real-time and non-destructive for agricultural phenotyping, but they must adhere to strict operational standards to ensure their safe use. Strict airspace regulations in many jurisdictions around the world and particularly in African countries due to impact of political instability and military governments on UAV deployment may prohibit their use or make them unfeasible in practice (Gago et al., 2015; Yang et al., 2017a; Ayamga et al., 2021). For instance, authorization from regulatory authorities, such as the air force, civil aviation and police, are required to undertake UAV flight campaigns, which mostly take time to be approved causing issues in time-critical data collection applications. According to Ayamga et al. (2021), in Africa, countries with regulations include Ghana, South Africa, Zimbabwe, Nigeria, Cameroon, Benin, Gabon, Senegal, Botswana, Namibia, Malawi, Tanzania, Zambia, Madagascar, Rwanda and Kenya. However, the lack of proper regulation and enforcement continues to limit the widespread adoption of drones. Unfortunately, these regulations combine to mean that most high-throughput techniques can only be implemented by multinational research institutions, even in those organizations, deployment of systems is limited to a few high-priority projects. Commitment of African governments and relevant stakeholders is crucial in the implementation and enforcement of regulations. The widespread deployment of drones stands to benefit farmers hence concerted effort need to be made to sustain its adoption by promoting public digital literacy on the technology, skill development for potential users and farmers on drone operation and developing the necessary policy framework with regulatory agencies to increase the safety and acceptability of using agricultural drones in Africa.

### 6.6 Weakness of phenotyping linkages

At the regional and continental levels, networking is a powerful tool for increasing scientific collaboration and fostering information sharing. There seems to be weak collaborations between the African plant science community and international phenotyping partners which hampers technological transfer and adoption. As high-throughput field phenotyping initiatives have started in Africa, there is a need to strengthen national and institutional efforts within the continent for the development and application of accurate and high-throughput field phenotyping capabilities. The West Africa field phenotyping network should be strengthened and better resourced to carry out their mandate. Similar initiatives such as the EMPHASIS (https://emphasis.plant-phenotyping.eu) should be experimented to provide a more practical use of the available phenotyping data.

The IPPN should spread its operations to Africa to develop programs and establish synergies geared towards face-lifting plant phenotyping projects in the continent. Again, African governments and their partners should invest in building a center of excellence or shared facilities for African plant scientists. Finally, a more urgent challenge is, however, that the international phenotyping community needs to bridge the gap between advanced economies and developing regions of the world such as Africa to benefit from the huge research efforts made internationally.

## 7 Concluding remarks and future perspectives

This review provides an overview of high-throughput field phenotyping and its implications for African crops. It highlights the prospects of emerging high-throughput phenotyping techniques and integrated sensor platforms for plant trait assessment for field phenotyping that could apply to African crops. High-throughput field phenotyping has superior advantages that facilitate quick, non-destructive, and high-throughput detection, thus overcoming the shortcomings of conventional approaches. The readiness and the potential adoption of high-throughput field phenotyping for practical implementation in Africa are of paramount interest and should be demonstrated.

Field phenotyping solutions of immediate to long-term feasibility for African crops will likely rely on a combination of available techniques or prototypes of low-cost sensors and imaging approaches to study crop performance. Manual methods dominate the field phenotyping ecosystem with only a few countries beginning to explore high-throughput techniques through digital and precision agriculture. Notably, high-throughput phenotyping cannot yet completely replace manual measurements but should be promoted. The implementation of high-throughput phenotyping in general, and low-cost methods for field evaluation, is still fraught with challenges in Africa. Challenges identified by this present review include the high upfront cost of the prototypical platforms, huge funding gap, lack of conceptual and technical capacity, lack of technology transfer infrastructure and methodological approaches, lack of phenotyping network on the continent and the needed legislation in some cases, amongst others.

Lack of financial resources, a problem pervasive in African countries needs to be tackled holistically. Public-private partnerships could support resolving these financial and investment challenges to foster political will. Although in some countries, this public-private drive is already being implemented through close collaboration between universities and agricultural research organizations, these efforts need to be stepped up. In parallel, African governments should dedicate enough funding, incentives, and tools to breeders to advance research and innovations regarding high-end plant breeding. We suggest that donor support to Africa for agriculture and food security research should also consider projects in modern plant phenotyping to cope with current and projected climate change.

This will open the possibility of investing more in current sensor and imaging technologies for field data collection and the use of cost-effective phenotyping technologies that are already available to increase the throughput, quantity and quality of phenotypic data. The wide range of applications for these phenotyping technologies makes them good candidates and feasible choices for adoption in Africa which hitherto were prohibitive in terms of cost and deployment. The advantages of improved sensor-platform integration have facilitated the development of complete phenotyping systems that can gather, integrate and store data for many subsystems concurrently in a structured, efficient and cost-effective way. Such platforms have been widely adopted by research groups in developed countries and are gradually adopted by plant breeders in Africa as the technology develops and the benefits are proven.

In addition to the adoption of high throughput field phenotyping approaches in African countries, precision agriculture will also greatly benefit and revitalize the establishment of closer interaction between breeders and farmers to develop protocols mutually for the optimal use of improved crop varieties. The tools and knowledge exchange are expected to spur a second green revolution to meet the agricultural challenges to feed the ever-increasing African population. In terms of advancing field crop phenotyping in Africa for agricultural crop sustainability, we propose that breeding priority should be given to the most important staple crops such as maize, wheat, yam, cassava, cowpea, sorghum, etc. These crops form the backbone for food security and hence their improvement is crucial in the wake of prevailing climate change and production constraints. We suggest that each country selects traits that are of high demand and relevance by farmers and consumers when designing breeding strategies. In parallel, high-throughput phenotyping should be incorporated into national agricultural research policies and prioritize the practical implementation of field phenotyping. By and large, these could be achieved when governmental and private sector participation, as well as financial support, is readily available.

To overcome the challenges with the deployment of phenotyping tools and the integration of software to deliver accurate data acquisition, processing, analysis and management, a multidisciplinary team of expert-level skills and competencies may be required. This will necessitate deliberate training and capacity improvement of African plant scientists and students in software engineering and computer science domains, including AI, demanding true interdisciplinary partnerships to provide meaningful results and inform decision-making, while addressing the issue of training cost and related risks. In this instance, we recommend technological adoption rather than complete technological development considering the financial constraints and the low-level expertise in software and equipment development. However, as the plant phenotyping industry develops the development of new technologies from scratch may be feasible in Africa.

Furthermore, we propose encouraging collaborations between the African plant science community with their international counterparts to foster collaborative research, effective technological transfer and adoption. This review recommends close collaboration with the IPPN and similar phenotyping networks to benefit from the unprecedented investments made in field phenotyping infrastructures globally. Consequently, crop scientists may leverage ground-breaking advancements in high-throughput field data collection, image analysis and data management. Efforts should be made to foster synergies among different African countries by establishing transnational interdisciplinary networks that incorporate expertise in all aspects of plant breeding.

To address the limited investments in science, technology and innovation (STI), a commitment for expanded and long-term funding of agricultural research and development is essential. At the policy and operational levels, barriers must be overcome to allow the smooth establishment of public-private partnerships for transformational change in research and demand-driven technologies for breeders and farmers. There is renewed interest both from private and public institutions in developed countries to support African agriculture. Hence, African agricultural institutions need to develop strategies and synergies that include building partnerships that must be implemented to tackle the challenges, especially in the face of climate change and food insecurity.

The widespread adoption of high-throughput field phenotyping techniques in African countries could only be made possible in plant breeding programs if it can be proven as something worthwhile in terms of genetic gains attained with resources invested. Hence, costs must be reasoned in relation to the precision, repeatability, heritability, cost per unit plot or trait, prevailing climatic and economic condition, etc., required in a particular phenotyping activity. Given what has been said, to ensure that such implementation of field phenotyping can be translated into yield gains, low-cost phenotyping tools must be adopted. On this basis, affordable, easy-to-handle, reliable tools, and phenotyping infrastructures for small to large-scale field phenotyping may become a strategic choice and pave the way for practical implementation. Such technologies applicable to phenotyping methodologies should be available soon due to the high demands and efforts by the phenotyping community in Africa.

## Author contributions

DC, NV, TW, FM, and MH provided the conceptualization of the manuscript. DC drafted, wrote, and edited the manuscript. NV, MC, AR, MM, TW, FM, and MH substantially reviewed the manuscript. TW, FM, and MH provided supervision. All authors read and approved the final manuscript. All authors contributed to the article.

## References

[B1] Aasen H. Burkart A. Bolten A. Bareth G. (2015). Generating 3D hyperspectral information with lightweight UAV snapshot cameras for vegetation monitoring: from camera calibration to quality assurance. ISPRS J. Photogrammetry Remote Sens. 108, 245–259. doi: 10.1016/j.isprsjprs.2015.08.002

[B2] Abay K. A. Abate G. T. Barrett C. B. Bernard T. (2019). Correlated non-classical measurement errors, ‘Second best’ policy inference, and the inverse size-productivity relationship in agriculture. J. Dev. Economics 139, 171–184. doi: 10.1016/j.jdeveco.2019.03.008

[B3] Abdelnabby M. Khalil T. (2023). Assessing precision agriculture applicability in agriculture sector in Egypt. Fayoum J. Agric. Res. Dev. 37 (1), 54–62. doi: 10.21608/fjard.2023.281055

[B4] Abdulai A. R. (2022). Toward digitalization futures in smallholder farming systems in Sub-Sahara Africa: A social practice proposal. Front. Sustain. Food Syst. 6. doi: 10.3389/fsufs.2022.866331

[B5] Abdulai A. R. Kc K. B. Fraser E. (2023). What factors influence the likelihood of rural farmer participation in digital agricultural services? experience from smallholder digitalization in Northern Ghana. Outlook Agric. 52 (1), 57–66. doi: 10.1177/00307270221144641 PMC13021113 41907093

[B6] Adhikari U. Nejadhashemi A. P. Woznicki S. A. (2015). Climate change and eastern Africa: a review of impact on major crops. Food Energy Secur. 4 (2), 110–132. doi: 10.1002/fes3.61

[B7] Affoh R. Zheng H. Dangui K. Dissani B. M. (2022). The impact of climate variability and change on food security in sub-saharan Africa: Perspective from panel data analysis. Sustainability 14 (2), 759. doi: 10.3390/su14020759

[B8] AGRA. (2019). A handbook of plant breeding: Case studies from Africa 2019. Available at: https://agra.org/wp-content/uploads/2022/09/Handbook-of-Plant-Breeding-Case-Studies-from-Africa-2019.pdf (Accessed July 15, 2023).

[B9] Ahmed H. G. M. D. Zeng Y. Fiaz S. Rashid A. R. (2023). “Applications of high-throughput phenotypic phenomics,” in Sustainable agriculture in the era of the OMICs revolution. Eds. Prakash C. S. Fiaz S. Nadeem M. A. Baloch F. S. Qayyum A. (Cham: Springer). doi: 10.1007/978-3-031-15568-0_6

[B10] Alabi T. R. Abebe A. T. Chigeza G. Fowobaje K. R. (2022). Estimation of soybean grain yield from multispectral high-resolution UAV data with machine learning models in West Africa. Remote Sens. Applications: Soc. Environ. 27, 100782. doi: 10.1016/j.rsase.2022.100782

[B11] Alemaw G. Agegnehu G. (2019). Precision agriculture and the need to introduce in Ethiopia. Ethiopian J. Agric. Sci. 29 (3), 139–158.

[B12] Andrade-Sanchez P. Gore M. A. Heun J. T. Thorp K. R. Carmo-Silva A. E. French A. N.. (2014). Development and evaluation of a field-based high-throughput phenotyping platform. Funct. Plant Biol. 41 (1), 68–79. doi: 10.1071/FP13126 32480967

[B13] Andrianto H. Suhardi S. Faizal A. (2017). “Measurement of chlorophyll content to determine nutrition deficiency in plants: A systematic literature review,” in 2017 International Conference on Information Technology Systems and Innovation, ICITSI 2017 - Proceedings, 2018-January. (Piscataway, New Jersey, United States: Institute of Electrical and Electronics Engineers (IEEE)), pp. 392–397. doi: 10.1109/ICITSI.2017.8267976

[B14] Appels R. Eversole K. Feuillet C. Keller B. Rogers J. Stein N.. (2018). Shifting the limits in wheat research and breeding using a fully annotated reference genome. Science 361 (6403). doi: 10.1126/science.aar7191 30115783

[B15] Araus J. L. Cairns J. E. (2014). Field high-throughput phenotyping: The new crop breeding frontier. Trends Plant Sci. 19 (1), 52–61. doi: 10.1016/j.tplants.2013.09.008 24139902

[B17] Araus J. L. Kefauver S. C. Vergara-Díaz O. Gracia-Romero A. Rezzouk F. Z. Segarra J.. (2022). Crop phenotyping in a context of global change: What to measure and how to do it. J. Integr. Plant Biol. 64 (2), 592–618. doi: 10.1111/jipb.13191 34807514

[B18] Araus J. L. Slafer G. A. Royo C. Serret M. D. (2008). Breeding for yield potential and stress adaptation in cereals. Crit. Rev. Plant Sci. 27 (6), 377–412. doi: 10.1080/07352680802467736

[B19] Asare-Bediako E. Taah K. J. van der Puije G. Amenorpe G. Appiah-Kubi A. Akuamoa-Boateng S. (2019). Evaluation of maize (Zea mays L.) genotypes for high grain yield and resistance to maize streak virus infections under diverse agro-ecological zones. Res. J. Plant Pathol. 2 (2), 11. doi: 10.36648/plantpathology.2.2.11

[B20] Atanga S. N. (2020). Digitalization of agriculture: how digital technology is transforming small-scale farming in Ghana. Agrarian, Food and Environmental Studies (AFES). A research paper submitted to Erasmus University in partial fulfilment of the requirements for the degree of Master of Arts in Development Studies at the International Institute of Social Studies.

[B22] Audebert A. Luquet D. Vadez V. Fonceka D. Kane N. A. (2022). Toward a regional field phenotyping network in West Africa. Available at: https://agritrop.cirad.fr/604058/2/ID604058.pdf.

[B23] Ayamga M. Tekinerdogan B. Kassahun A. (2021). Exploring the challenges posed by regulations for the use of drones in agriculture in the African context. Land 10 (2), 164. doi: 10.3390/land10020164

[B24] Badu-Apraku B. Fakorede M. A. Nelimor C. Osuman A. S. Bonkoungou T. O. Muhyideen O.. (2023). Recent advances in breeding maize for drought, heat and combined heat and drought stress tolerance in sub-saharan Africa. CABI Rev. Volume 2023. doi: 10.1079/cabireviews.2023.0011

[B25] Bahn R. A. Yehya A. A. K. Zurayk R. (2021). Digitalization for sustainable agri-food systems: Potential, status, and risks for the MENA Region. Sustainability 13, 3223. doi: 10.3390/su13063223

[B26] Bai G. Ge Y. Scoby D. Leavitt B. Stoerger V. Kirchgessner N.. (2019). NU-Spidercam: a large-scale, cable-driven, integrated sensing and robotic system for advanced phenotyping, remote sensing, and agronomic research. Comput. Electron. Agric. 160, 71–81. doi: 10.1016/j.compag.2019.03.009

[B1004] Bai G. Ge Y. Hussain W. Baenziger P. S. Graef G. (2016). A multi-sensor system for high throughput field phenotyping in soybean and wheat breeding. Computers and Electronics in Agriculture 128, 181–192. doi: 10.1016/j.compag.2016.08.021

[B27] Bascon M. V. Nakata T. Shibata S. Takata I. Kobayashi N. Kato Y.. (2022). Estimating yield-related traits using UAV-derived multispectral images to improve rice grain yield prediction. Agric. (Switzerland) 12 (8), 1141. doi: 10.3390/agriculture12081141

[B28] Baumüller H. Kah M. M. (2019). Going digital: Harnessing the power of emerging technologies for the transformation of Southern African agriculture. Transforming Agriculture in Southern Africa: Constraints, Technologies, Policies and Policies and Processes (Routledge: Taylor and Francis Inc., Milton Park, Abingdon in the United Kingdom), 179–187.

[B29] Belmahi M. Hanchane M. Krakauer N. Y. Kessabi R. Bouayad H. Mahjoub A.. (2023). Analysis of relationship between grain yield and NDVI from MODIS in the fez-meknes region, Morocco. Remote Sens. 15 (11), 2707. doi: 10.3390/rs15112707

[B30] Ben M’Barek S. Laribi M. Kouki H. Castillo D. Araar C. Nefzaoui M.. (2022). Phenotyping mediterranean durum wheat landraces for resistance to zymoseptoria tritici in Tunisia. Genes 13 (2), 355. doi: 10.3390/genes13020355 35205399 PMC8872163

[B31] Beyene A. N. Zeng H. Wu B. Zhu L. Gebremicael T. G. Zhang M.. (2022). Coupling remote sensing and crop growth model to estimate national wheat yield in Ethiopia. Big Earth Data 6 (1), 18–35. doi: 10.1080/20964471.2020.1837529

[B32] Bian J. Zhang Z. Chen J. Chen H. Cui C. Li X.. (2019). Simplified evaluation of cotton water stress using high resolution unmanned aerial vehicle thermal imagery. Remote Sens. 11 (3), 267. doi: 10.3390/rs11030267

[B33] Bijaber N. El Hadani D. Saidi M. Svoboda M. D. Wardlow B. D. Hain C. R.. (2018). Developing a remotely sensed drought monitoring indicator for Morocco. Geosciences (Switzerland) 8 (2), 55. doi: 10.3390/geosciences8020055 PMC7427818 32802481

[B34] Bjornlund V. Bjornlund H. Van Rooyen A. F. (2020). Why agricultural production in sub-Saharan Africa remains low compared to the rest of the world–a historical perspective. Int. J. Water Resour. Development 36 (sup1), S20–S53, 1–34. doi: 10.1080/07900627.2020.1739512

[B35] Blancon J. Dutartre D. Tixier M. H. Weiss M. Comar A. Praud S.. (2019). A high-throughput model-assisted method for phenotyping maize green leaf area index dynamics using unmanned aerial vehicle imagery. Front. Plant Sci. 10. doi: 10.3389/fpls.2019.00685 PMC6568052 31231403

[B36] Blein R. Soule B. G. Dupaigre B. F. Yerima B. (2008). “Agricultural potential of west Africa,” in Economic community of West African States (ECOWAS) (Foundation pour l’agriculture et la ruralité dans le monde (FARM), 118. Available at: https://old.fondation-farm.org/IMG/pdf/potentialites_rapport_ang_mp.pdf.

[B37] Bongomin O. Lamo J. Guina J. Okello C. Ocen G. Obura M.. (2022). Applications of Drones and Image Analytics in Field Phenotyping: A Potential Breakthrough in Uganda's Agricultural Research. SSRN Electron. J. Available at SSRN 4158755. 10.2139/ssrn.4158755.

[B1006] Bontpart T. Concha C. Giuffrida M. V. Robertson I. Admkie K. Degefu T.. (2020). Affordable and robust phenotyping framework to analyse root system architecture of soil‐grown plants. Plant J. 103 (6), 2330–2343. doi: 10.1111/tpj.14877 32530068

[B38] Born L. Chirinda N. Mabaya E. Afun-Ogidan O. Girvetz E. H. Jarvis A.. (2021). Digital agriculture profile: South Africa (FAO). Available at: http://www.fao.org/3/cb2506en/CB2506EN.pdf (Accessed August 4, 2023).

[B40] Bouras E. H. Jarlan L. Er-Raki S. Albergel C. Richard B. Balaghi R.. (2020). Linkages between rainfed cereal production and agricultural drought through remote sensing indices and a land data assimilation system: A case study in Morocco. Remote Sens. 12 (24), 4018. doi: 10.3390/rs12244018

[B41] Bradshaw C. D. Pope E. Kay G. Davie J. C. Cottrell A. Bacon J.. (2022). Unprecedented climate extremes in South Africa and implications for maize production. Environ. Res. Lett. 17 (8), 084028. doi: 10.1088/1748-9326/ac816d

[B42] Brewer K. Clulow A. Sibanda M. Gokool S. Naiken V. Mabhaudhi T. (2022b). Predicting the chlorophyll content of maize over phenotyping as a proxy for crop health in smallholder farming systems. Remote Sens. 14 (3), 518. doi: 10.3390/rs14030518

[B43] Brewer K. Clulow A. Sibanda M. Gokool S. Odindi J. Mutanga O.. (2022a). Estimation of maize foliar temperature and stomatal conductance as indicators of water stress based on optical and thermal imagery acquired using an unmanned aerial vehicle (UAV) platform. Drones 6 (7), 169. doi: 10.3390/drones6070169

[B44] Brocks S. Bareth G. (2018). Estimating barley biomass with crop surface models from oblique RGB imagery. Remote Sens. 10 (2), 268. doi: 10.3390/rs10020268

[B45] Buchaillot M. L. Gracia-Romero A. Vergara-Diaz O. Zaman-Allah M. A. Tarekegne A. Cairns J. E.. (2019). Evaluating maize genotype performance under low nitrogen conditions using RGB UAV phenotyping techniques. Sensors (Switzerland) 19 (8), 1815. doi: 10.3390/s19081815 PMC6514658 30995754

[B47] Burke M. Lobell D. B. (2017). Satellite-based assessment of yield variation and its determinants in smallholder African systems. Proc. Natl. Acad. Sci. United States America 114 (9), 2189–2194. doi: 10.1073/pnas.1616919114 PMC5338538 28202728

[B1003] Burnette M. Kooper R. Maloney J. D. Rohde G. S. Terstriep J. A. Willis C.. (2018). TERRA-REF data processing infrastructure. In Proceedings of the Practice and Experience on Advanced Research Computing, pp. 1–7. doi: 10.1145/3219104.3219152

[B48] Busemeyer L. Mentrup D. Möller K. Wunder E. Alheit K. Hahn V.. (2013). Breedvision - A multi-sensor platform for non-destructive field-based phenotyping in plant breeding. Sensors (Switzerland) 13 (3), 2830–2847. doi: 10.3390/s130302830 PMC3658717 23447014

[B49] Buthelezi S. Mutanga O. Sibanda M. Odindi J. Clulow A. D. Chimonyo V. G.. (2023). Assessing the prospects of remote sensing maize leaf area index using UAV-derived multi-spectral data in smallholder farms across the growing season. Remote Sens. 15 (6), 1597. doi: 10.3390/rs15061597

[B50] Camino C. González-Dugo V. Hernández P. Sillero J. C. Zarco-Tejada P. J. (2018). Improved nitrogen retrievals with airborne-derived fluorescence and plant traits quantified from VNIR-SWIR hyperspectral imagery in the context of precision agriculture. Int. J. Appl. Earth Observation Geoinformation 70, 105–117. doi: 10.1016/j.jag.2018.04.013

[B1007] Casadesús J. Villegas D. (2014). Conventional digital cameras as a tool for assessing leaf area index and biomass for cereal breeding. J. of Integr. Plant Biol. 56 (1), 7–14. doi: 10.1111/jipb.12117 24330531

[B51] Cavaco A. M. Utkin A. B. da Silva J. M. Guerra R. (2022). Making sense of light: The use of optical spectroscopy techniques in plant sciences and agriculture. Appl. Sci. (Switzerland) 12 (3), 997. doi: 10.3390/app12030997

[B52] Chaerle L. Van Der Straeten D. (2000). Imaging techniques and the early detection of plant stress. Trends Plant Sci. 5 (11), 495–501. doi: 10.1016/S1360-1385(00)01781-7 11077259

[B53] Chapman S. C. Merz T. Chan A. Jackway P. Hrabar S. Dreccer M. F.. (2014). Pheno-copter: A low-altitude, autonomous remote-sensing robotic helicopter for high-throughput field-based phenotyping. Agronomy 4 (2), 279–301. doi: 10.3390/agronomy4020279

[B54] Chapu I. Okello D. K. Okello R. C. O. Odong T. L. Sarkar S. Balota M. (2022). Exploration of alternative approaches to phenotyping of late leaf spot and groundnut rosette virus disease for groundnut breeding. Front. Plant Sci. 13. doi: 10.3389/fpls.2022.912332 PMC9238324 35774822

[B55] Chawade A. Van Ham J. Blomquist H. Bagge O. Alexandersson E. Ortiz R. (2019). High-throughput field-phenotyping tools for plant breeding and precision agriculture. Agronomy 9 (5), 258. doi: 10.3390/agronomy9050258

[B56] Chen J. M. Black T. A. (1992). Defining leaf area index for non-flat leaves. Plant Cell Environ. 15 (4), 421–429. doi: 10.1111/j.1365-3040.1992.tb00992.x

[B57] Chivasa W. Mutanga O. Biradar C. (2017). Application of remote sensing in estimating maize grain yield in heterogeneous african agricultural landscapes: A review. Int. J. Remote Sens. 38 (23), 6816–6845. doi: 10.1080/01431161.2017.1365390

[B58] Chivasa W. Mutanga O. Biradar C. (2020). UAV-based multispectral phenotyping for disease resistance to accelerate crop improvement under changing climate conditions. Remote Sens. 12 (15), 2445. doi: 10.3390/RS12152445

[B59] Choukr-Allah R. Rao N. K. Hirich A. Shahid M. Alshankiti A. Toderich K.. (2016). Quinoa for marginal environments: toward future food and nutritional security in MENA and Central Asia regions. Front. Plant Sci. 7. doi: 10.3389/fpls.2016.00346 PMC4810016 27066019

[B60] Comar A. Burger P. De Solan B. Baret F. Daumard F. Hanocq J. F. (2012). A semi-automatic system for high throughput phenotyping wheat cultivars in-field conditions: Description and first results. Funct. Plant Biol. 39 (11), 914–924. doi: 10.1071/FP12065 32480841

[B61] Condorelli G. E. Maccaferri M. Newcomb M. Andrade-Sanchez P. White J. W. French A. N.. (2018). Corrigendum: Comparative aerial and ground based high throughput phenotyping for the genetic dissection of NDVI as a proxy for drought adaptive traits in durum wheat (Front. Plant Sci. 9, 893, 10.3389/fpls.2018.00893). Front. Plant Sci. 9. doi: 10.3389/fpls.2018.01885 PMC6028805 29997645

[B62] Costa J. M. Grant O. M. Chaves M. M. (2013). Thermography to explore plant-environment interactions. J. Exp. Bot. 64 (13), 564–584. doi: 10.1093/jxb/ert029 23599272

[B63] Costa J. M. Marques da Silva J. Pinheiro C. Barón M. Mylona P. Centritto M.. (2019). Opportunities and limitations of crop phenotyping in Southern European Countries. Front. Plant Sci. 10. doi: 10.3389/fpls.2019.01125 PMC6774291 31608085

[B64] Cozzolino D. (2014). Use of infrared spectroscopy for in-field measurement and phenotyping of plant properties: Instrumentation, data analysis, and examples. Appl. Spectrosc. Rev. 49 (7), 564–584. doi: 10.1080/05704928.2013.878720

[B65] Crain J. L. Wei Y. Barker J. Thompson S. M. Alderman P. D. Reynolds M.. (2016). Development and deployment of a portable field phenotyping platform. Crop Sci. 56 (3), 965–975. doi: 10.2135/cropsci2015.05.0290

[B66] Crossa J. Pérez-Rodríguez P. Cuevas J. Montesinos-López O. Jarquín D. de los Campos G.. (2017). Genomic selection in plant breeding: Methods, models, and perspectives. Trends Plant Sci. 22 (11), 961–975. doi: 10.1016/j.tplants.2017.08.011 28965742

[B67] Dammer K. H. Dworak V. Selbeck J. (2016). On-the-go phenotyping in field potatoes using camera vision. Potato Res. 59 (2), 113–127. doi: 10.1007/s11540-016-9315-y

[B68] Daniel I. O. Atinsola K. O. Ajala M. O. Popoola A. R. (2016). Phenotyping a tomato breeding population by manual field evaluation and digital imaging analysis. Int. J. Plant Breed. Genet. 11 (1), 19–24. doi: 10.3923/ijpbg.2017.19.24

[B69] Danzi D. Briglia N. Petrozza A. Summerer S. Povero G. Stivaletta A.. (2019). Can high throughput phenotyping help food security in the mediterranean area? Front. Plant Sci. 10. doi: 10.3389/fpls.2019.00015 PMC6355677 30740116

[B70] Debalke D. B. Abebe J. T. (2022). Maize yield forecast using GIS and remote sensing in Kaffa Zone, South-West Ethiopia. Environ. Syst. Res. 11 (1), 1. doi: 10.1186/s40068-022-00249-5

[B71] Debesa G. Gebre S. L. Melese A. Regassa A. Teka S. (2020). GIS and remote sensing-based physical land suitability analysis for major cereal crops in Dabo Hana district, South-West Ethiopia. Cogent Food Agric. 6 (1), 1780100. doi: 10.1080/23311932.2020.1780100

[B73] Deery D. Jimenez-Berni J. Jones H. Sirault X. Furbank R. (2014). Proximal remote sensing buggies and potential applications for field-based phenotyping. Agronomy 4 (3), 875. doi: 10.3390/agronomy4030349

[B72] Deery D. M. Rebetzke G. J. Jimenez-Berni J. A. Bovill W. D. James R. A. Condon A. G.. (2019). Evaluation of the phenotypic repeatability of canopy temperature in wheat using continuous-terrestrial and airborne measurements. Front. Plant Sci. 10. doi: 10.3389/fpls.2019.00875 PMC6629910 31338102

[B74] Degila J. Sodedji F. A. K. Avakoudjo H. G. G. Tahi S. P. G. Houetohossou S. C. A. Honfoga A. C.. (2023). Digital agriculture policies and strategies for innovations in the agri-food systems – cases of five West African Countries. Sustainability 15 (12), 9192. doi: 10.3390/su15129192

[B75] Dingkuhn M. Sow A. Manneh B. Radanielina T. Raboin L. M. Dusserre J.. (2015). Field phenomics for response of a rice diversity panel to ten environments in Senegal and Madagascar. 1. Plant phenological traits. Field Crops Res. 183, 342–355. doi: 10.1016/j.fcr.2015.07.027

[B77] Duvallet M. Dumas P. Makowski D. Boé J. del Villar P. M. Ben-Ari T. (2021). Rice yield stability compared to major food crops in West Africa. Environ. Res. Lett. 16 (12), 124005. doi: 10.1088/1748-9326/ac343a

[B79] Ejikeme J. O. Ojiako J. C. Onwuzuligbo C. U. Ezeh F. C. (2017). Enhancing food security in Anambra state, Nigeria using remote sensing data. Environ. Rev. 6 (1), 27–44. Available at: http://erjournal.net/index.php/erjournal/article/viewFile/33/pdf4

[B80] Elmetwalli A. H. Mazrou Y. S. A. Tyler A. N. Hunter P. D. Elsherbiny O. Yaseen Z. M.. (2022). Assessing the efficiency of remote sensing and machine learning algorithms to quantify wheat characteristics in the nile delta region of Egypt. Agriculture 12 (3), 332. doi: 10.3390/agriculture12030332

[B1005] Elmetwalli A. H. El-Hendawy S. Al-Suhaibani N. Alotaibi M. Tahir M. U. Mubushar M.. (2020). Potential of hyperspectral and thermal proximal sensing for estimating growth performance and yield of soybean exposed to different drip irrigation regimes under arid conditions. Sensors 20 (22), 6569. doi: 10.3390/s20226569 33213009 PMC7698533

[B81] Elsafty A. Atallah A. (2022). Factors influencing precision agriculture tools or technologies adoption in Egypt. Business Manage. Stud. 8 (2). doi: 10.11114/bms.v8i2.5598

[B82] Elsayed S. Elhoweity M. Ibrahim H. H. Dewir Y. H. Migdadi H. M. Schmidhalter U. (2017). Thermal imaging and passive reflectance sensing to estimate the water status and grain yield of wheat under different irrigation regimes. Agric. Water Manage. 189, 98–110. doi: 10.1016/j.agwat.2017.05.001

[B83] El-Shirbeny M. A. Ali A. Saleh N. H. (2014). Crop water requirements in Egypt using remote sensing techniques. J. Agric. Chem. Environ. 3 (2), 57–65. doi: 10.4236/jacen.2014.32B010

[B84] Epule T. E. Chehbouni A. Dhiba D. (2022). Recent patterns in maize yield and harvest area across Africa. Agronomy 12 (2), 374. doi: 10.3390/agronomy12020374

[B85] Erdle K. Mistele B. Schmidhalter U. (2013). Spectral assessments of phenotypic differences in spike development during grain filling affected by varying N supply in wheat. J. Plant Nutr. Soil Sc. 176, 952–963. doi: 10.1002/jpln.201300247

[B86] Fahrner S. Janni M. Pieruschka R. Vincenz-Donnelly L. von Gillhaussen P. (2021). Global plant phenotyping survey 2020/21 (Zenodo). doi: 10.5281/zenodo.4723409

[B88] FAO. (2011). Evolving a plant breeding and seed system in sub-Saharan Africa in an era of donor dependence. A report for the Global Partnership Initiative for Plant Breeding Capacity Building (GIPB) of the Food and Agricultural Organisation. FAO plant production and protection paper 210, pp. 21–30. Available at: https://www.fao.org/3/at535e/at535e.pdfb.

[B89] FAO. (2015). FAOSTAT. Food and agricultural organisation of the United Nations. Available at: http://www.fao.org/faostat/en/#data/.

[B87] FAO. (2022). FAOSTAT. Database of the food and agriculture organization united nations. Available at: www.fao.org/faostat/en/.

[B1001] FAOSTAT. (2022). Database of the Food and Agriculture Organization of the United Nations, Rome, Italy. Available at: https://www.fao.org/faostat/en/. Accessed on August 12, 2023.

[B91] FAOSTAT. (2018a). Database of the food and agriculture organization united nations. Available at: www.fao.org/faostat/en/.

[B92] FAOSTAT. (2018b). Morocco production in 2018. Available at: https://www.fao.org/faostat/en/#data/QCL.

[B1002] FAOSTAT. (2016). FAOSTAT statistical database. Food and Agriculture Organization of the United Nations, Rome, Italy. Accessed on August 12, 2023.

[B93] Feng L. Chen S. Zhang C. Zhang Y. He Y. (2021). A comprehensive review on recent applications of unmanned aerial vehicle remote sensing with various sensors for high-throughput plant phenotyping. Comput. Electron. Agric. 182, 106033. doi: 10.1016/j.compag.2021.106033

[B94] Feng W. Yao X. Zhu Y. Tian Y. C. Cao W. X. (2008). Monitoring leaf nitrogen status with hyperspectral reflectance in wheat. Eur. J. Agron. 28 (3), 394–404. doi: 10.1016/j.eja.2007.11.005

[B95] Fernández-Calleja M. Monteagudo A. Casas A. M. Boutin C. Pin P. A. Morales F.. (2020). Rapid on-site phenotyping via field fluorimeter detects differences in photosynthetic performance in a hybrid—parent barley germplasm set. Sensors (Switzerland) 20 (5), 1486. doi: 10.3390/s20051486 PMC7085516 32182722

[B96] Fiorani F. Schurr U. (2013). Future scenarios for plant phenotyping. Annu. Rev. Plant Biol. 64, 267–291. doi: 10.1146/annurev-arplant-050312-120137 23451789

[B97] Fisher M. Abate T. Lunduka R. W. Asnake W. Alemayehu Y. Madulu R. B. (2015). Drought tolerant maize for farmer adaptation to drought in sub-Saharan Africa: Determinants of adoption in eastern and southern Africa. Climatic Change 133, 283–299. doi: 10.1007/s10584-015-1459-2

[B98] Fitzgerald G. J. Rodriguez D. Christensen L. K. Belford R. Sadras V. O. Clarke T. R. (2006). Spectral and thermal sensing for nitrogen and water status in rainfed and irrigated wheat environments. Precis. Agric. 7 (4), 233–248. doi: 10.1007/s11119-006-9011-z

[B99] Fritsche-Neto R. Borém A. (2015). Phenomics: How next-generation phenotyping is revolutionizing plant breeding. In Phenomics: How Next-Generation Phenotyping is Revolutionizing Plant Breeding. (Switzerland: Springer International Publishing). doi: 10.1007/978-3-319-13677-6

[B102] Furbank R. T. Jimenez-Berni J. A. George-Jaeggli B. Potgieter A. B. Deery D. M. (2019). Field crop phenomics: enabling breeding for radiation use efficiency and biomass in cereal crops. New Phytol. 223 (4), 1714–1727. doi: 10.1111/nph.15817 30937909

[B103] Gago J. Douthe C. Coopman R. E. Gallego P. P. Ribas-Carbo M. Flexas J.. (2015). UAVs challenge to assess water stress for sustainable agriculture. Agric. Water Manage. 153, 9–19. doi: 10.1016/j.agwat.2015.01.020

[B104] Gano B. Dembele J. S. B. Ndour A. Luquet D. Beurier G. Diouf D.. (2021). Adaptation responses to early drought stress of West Africa sorghum varieties. Agronomy 11 (5), 850. doi: 10.3390/agronomy11050850

[B105] Garriga M. Romero-Bravo S. Estrada F. Escobar A. Matus I. A. del Pozo A.. (2017). Assessing wheat traits by spectral reflectance: Do we really need to focus on predicted trait-values or directly identify the elite genotypes group? Front. Plant Sci. 8. doi: 10.3389/fpls.2017.00280 PMC5343032 28337210

[B106] Gedil M. Menkir A. (2019). An integrated molecular and conventional breeding scheme for enhancing genetic gain in maize in Africa. Front. Plant Sci. 10. doi: 10.3389/fpls.2019.01430 PMC6851238 31781144

[B107] Gibbs J. A. Pound M. French A. P. Wells D. M. Murchie E. Pridmore T. (2017). Approaches to three-dimensional reconstruction of plant shoot topology and geometry. Funct. Plant Biol. 44 (1), 62–75. doi: 10.1071/FP16167 32480547

[B108] Gobezie T. B. Biswas A. (2023). The need for streamlining precision agriculture data in Africa. Precis. Agric. 24 (1), 375–383. doi: 10.1007/s11119-022-09928-w

[B109] Godfray H. C. J. Robinson S. (2015). Contrasting approaches to projecting long-run global food security. Oxf Rev. Economic Policy 31 (1), 26–44. doi: 10.1093/oxrep/grv006

[B110] Gokool S. Mahomed M. Kunz R. Clulow A. Sibanda M. Naiken V.. (2023). Crop monitoring in smallholder farms using unmanned aerial vehicles to facilitate precision agriculture practices: a scoping review and bibliometric analysis. Sustainability 15 (4), 3557. doi: 10.3390/su15043557

[B111] Gonzalez-Dugo V. Hernandez P. Solis I. Zarco-Tejada P. J. (2015). Using high-resolution hyperspectral and thermal airborne imagery to assess physiological condition in the context of wheat phenotyping. Remote Sens. 7 (10), 13586–13605. doi: 10.3390/rs71013586

[B112] Gourlay S. Kilic T. Lobell D. (2017). Could the debate be over? Errors in farmer-reported production and their implications for the inverse scale-productivity relationship in Uganda. Technical Report, Policy Research Working Paper No. 8192. (Washington, D C: World Bank). doi: 10.1596/1813-9450-8192

[B113] Gracia-Romero A. Kefauver S. C. Vergara-Díaz O. Hamadziripi E. Zaman-Allah M. A. Thierfelder C.. (2020). Leaf versus whole-canopy remote sensing methodologies for crop monitoring under conservation agriculture: a case of study with maize in Zimbabwe. Sci. Rep. 10 (1), 16008. doi: 10.1038/s41598-020-73110-3 32994539 PMC7524805

[B114] Gracia-Romero A. Vergara-Díaz O. Thierfelder C. Cairns J. E. Kefauver S. C. Araus J. L. (2018). Phenotyping conservation agriculture management effects on ground and aerial remote sensing assessments of maize hybrids performance in Zimbabwe. Remote Sens. 10 (2), 349. doi: 10.3390/rs10020349 PMC7340492 32704486

[B117] Guo W. Fukatsu T. Ninomiya S. (2015). Automated characterization of flowering dynamics in rice using field-acquired time-series RGB images. Plant Methods 11 (1), 1–15. doi: 10.1186/s13007-015-0047-9 25705245 PMC4336727

[B116] Guo Q. Wu F. Pang S. Zhao X. Chen L. Liu J.. (2018). Crop 3D—a LiDAR based platform for 3D high-throughput crop phenotyping. Sci. China Life Sci. 61 (3), 328–339. doi: 10.1007/s11427-017-9056-0 28616808

[B118] Hall O. Dahlin S. Marstorp H. Archila Bustos M. F. Öborn I. Jirström M. (2018). Classification of maize in complex smallholder farming systems using UAV imagery. Drones 2 (3), 22. doi: 10.3390/drones2030022

[B119] Hamidi S. Benabdeljalil N. (2013). National innovation systems: The Moroccan case. Proc. - Soc. Behav. Sci. 75, 119–128. doi: 10.1016/J.SBSPRO.2013.04.014

[B120] Hassan M. A. Yang M. Fu L. Rasheed A. Zheng B. Xia X.. (2019). Accuracy assessment of plant height using an unmanned aerial vehicle for quantitative genomic analysis in bread wheat. Plant Methods 15 (1), 1–12. doi: 10.1186/s13007-019-0419-7 31011362 PMC6463666

[B121] Hedley C. (2015). The role of precision agriculture for improved nutrient management on farms. J. Sci. Food Agric. 95 (1), 12–19. doi: 10.1002/jsfa.6734 24816925

[B122] Henri A. O. (2019). Heavily indebted poor countries initiative (HIPC), debt relief, economic stability and economic growth in Africa. Economic Change Restructuring 52, 89–121. doi: 10.1007/s10644-017-9218-1

[B123] Hirich A. Rafik S. Rahmani M. Fetouab A. Azaykou F. Filali K.. (2021). Development of quinoa value chain to improve food and nutritional security in rural communities in Rehamna, Morocco: lessons learned and perspectives. Plants 10 (2), 301. doi: 10.3390/plants10020301 33562429 PMC7915470

[B124] Holman F. (2020). Development and evaluation of unmanned aerial vehicles for high throughput phenotyping of field-based wheat trials. (Doctoral dissertation, King's College London). Available at: https://repository.rothamsted.ac.uk/item/97q41/development-and-evaluation-of-unmanned-aerial-vehicles-for-high-throughput-phenotyping-of-field-based-wheat-trials

[B125] Holman F. H. Riche A. B. Michalski A. Castle M. Wooster M. J. Hawkesford M. J. (2016). High throughput field phenotyping of wheat plant height and growth rate in field plot trials using UAV based remote sensing. Remote Sens. 8 (12), 1031. doi: 10.3390/rs8121031

[B126] ICARDA phenotyping platforms in Morocco. Available at: https://www.cgiar.org/news-events/news/icardas-phenotyping-facilities-a-game-changing-solution-for-abiotic-stress-tolerance-in-crops/.

[B127] Ifeanyieze F. O. Ikehi M. E. Isiwu E. (2014). Techniques in utilizing remote sensor technology for precision crop production by farmers as climate change adaptation strategy in Nigeria. Agric. Sci. 5 (14), 1476. doi: 10.4236/as.2014.514158

[B128] Iizumi T. Sakai T. (2020). The global dataset of historical yields for major crops 1981–2016. Sci. Data 7 (1), 97. doi: 10.1038/s41597-020-0433-7 32198349 PMC7083933

[B131] IPPN. (2016). The international plant phenotyping network. In: Survey 2016. Available at: http://www.plant-phenotyping.org/lw_resource/datapool/systemfiles/elements/files/ba090795-026e-11e7-8c78-dead53a91d31/live/document/Survey_2016_final_2.pdf (Accessed June 20, 2023).

[B130] IPPN. (2020). Survey. Available at: https://globalplantcouncil.org/global-plant-phenotyping-survey-2020-21/.

[B132] Iseki K. Matsumoto R. (2019). Non-destructive shoot biomass evaluation using a handheld NDVI sensor for field-grown staking Yam (Dioscorea rotundata Poir.). Plant Production Sci. 22 (2), 301–310. doi: 10.1080/1343943X.2018.1540278

[B133] Izuogu C. U. Njoku L. C. Olaolu M. O. Kadurumba P. C. Azuamairo G. C. Agou G. D. (2023). A review of the digitalization of agriculture in Nigeria. J. Agric. Extension 27 (2), 47–64. doi: 10.4314/jae.v27i2.5

[B134] Jabir B. Falih N. (2020). Digital agriculture in Morocco, opportunities and challenges. In Proceedings of the 2020 6th International Conference on Optimization and Applications (ICOA), Beni Mellal, Morocco. (Piscataway, New Jersey, United States: Institute of Electrical and Electronics Engineers (IEEE)), 1–5, IEEE. doi: 10.1109/ICOA49421.2020.9094450

[B135] Jimenez-Berni J. A. Deery D. M. Rozas-Larraondo P. Condon A. T. G. Rebetzke G. J. James R. A.. (2018). High throughput determination of plant height, ground cover, and above-ground biomass in wheat with LiDAR. Front. Plant Sci. 9. doi: 10.3389/fpls.2018.00237 PMC5835033 29535749

[B136] Kassim Y. B. Oteng-Frimpong R. Puozaa D. K. Sie E. K. Abdul Rasheed M. Abdul Rashid I.. (2022). High-throughput plant phenotyping (HTPP) in resource-constrained research programs: A working example in Ghana. Agronomy 12 (11), 2733. doi: 10.3390/agronomy12112733

[B137] Kavhiza N. J. Zargar M. Prikhodko S. I. Pakina E. N. Murtazova K. M. S. Nakhaev M. R. (2022). Improving crop productivity and ensuring food security through the adoption of genetically modified crops in sub-saharan Africa. Agronomy 12 (2), 439. doi: 10.3390/agronomy12020439

[B138] Kefauver S. C. El-Haddad G. Vergara-Diaz O. Araus J. L. (2015). RGB picture vegetation indexes for high-throughput phenotyping platforms (HTPPs). Remote Sens. agriculture ecosystems hydrology XVII 9637, 82–90. SPIE. doi: 10.1117/12.2195235

[B139] Kidane Y. G. Hailemariam B. N. Mengistu D. K. Fadda C. Pè M. E. Dell’Acqua M. (2017). Genome-wide association study of Septoria tritici blotch resistance in Ethiopian durum Wheat Landraces. Front. Plant Sci. 8. doi: 10.3389/fpls.2017.01586 PMC5603693 28959268

[B140] Kim J. Y. (2020). Roadmap to high throughput phenotyping for plant breeding. J. Biosyst. Eng. 45, 43–55. doi: 10.1007/s42853-020-00043-0

[B141] Kipp S. Mistele B. Schmidhalter U. (2014). Identification of stay-green and early senescence phenotypes in high-yielding winter wheat, and their relationship to grain yield and grain protein concentration using high-throughput phenotyping techniques. Funct. Plant Biol. 41 (3), 227–235. doi: 10.1071/FP13221 32480983

[B142] Kirchgessner N. Liebisch F. Yu K. Pfeifer J. Friedli M. Hund A.. (2017). The ETH field phenotyping platform FIP: A cable-suspended multi-sensor system. Funct. Plant Biol. 44 (1), 154–168. doi: 10.1071/FP16165 32480554

[B143] Knox J. Hess T. Daccache A. Wheeler T. (2012). Climate change impacts on crop productivity in Africa and South Asia. Environ. Res. Lett. 7 (3), 034032. doi: 10.1088/1748-9326/7/3/034032

[B144] Kobo H. I. Abu-Mahfouz A. M. Hancke G. P. (2017). A survey on software-defined wireless sensor networks: Challenges and design requirements. IEEE Access 5, 1872–1899. doi: 10.1109/ACCESS.2017.2666200

[B145] Kotikot S. M. Onywere S. M. (2015). Application of GIS and remote sensing techniques in frost risk mapping for mitigating agricultural losses in the Aberdare ecosystem, Kenya. Geocarto Int. 30 (1), 104–121. doi: 10.1080/10106049.2014.965758

[B146] Kpienbaareh D. Kansanga M. Luginaah I. (2019). Examining the potential of open-source remote sensing for building effective decision support systems for precision agriculture in resource-poor settings. GeoJournal 84 (6), 1481–1497. doi: 10.1007/s10708-018-9932-x

[B147] Kumar D. Kushwaha S. Delvento C. Liatukas Ž. Vivekanand V. Svensson J. T.. (2020). Affordable phenotyping of winter wheat under field and controlled conditions for drought tolerance. Agronomy 10 (6), 882. doi: 10.3390/agronomy10060882

[B148] Laachrate H. Fadil A. Ghafiri A. (2020). Drought monitoring of some Moroccan agricultural areas: Settat and Meknes using remote sensing techniques and a soil moisture-based drought index. In Proceedings-2020 IEEE International Conference of Moroccan Geomatics, MORGEO 2020. doi: 10.1109/Morgeo49228.2020.9121917

[B149] Langemeier M. Boehlje M. (2021). What will be the capabilities and skills needed to manage the farm of the future? Farmdoc Dly. 11, 1–4. Department of Agricultural and Consumer Economics, University of Illinois at Urbana-Champaign.

[B150] Leakey R. R. Tientcheu Avana M. L. Awazi N. P. Assogbadjo A. E. Mabhaudhi T. Hendre P. S.. (2022). The future of food: Domestication and commercialization of indigenous food crops in Africa over the third decade, (2012–2021). Sustainability 14 (4), 2355. doi: 10.3390/su14042355

[B151] Li D. Quan C. Song Z. Li X. Yu G. Li C.. (2021). High-throughput plant phenotyping platform (HT3P) as a novel tool for estimating agronomic traits from the lab to the field. Front. Bioengineering Biotechnol. 8. doi: 10.3389/fbioe.2020.623705 PMC7838587 33520974

[B152] Li L. Zhang Q. Huang D. (2014). A review of imaging techniques for plant phenotyping. Sensors (Switzerland) 14 (11), 20078–20111. doi: 10.3390/s141120078 PMC4279472 25347588

[B153] Liu T. Li R. Jin X. Ding J. Zhu X. Sun C.. (2017). Evaluation of seed emergence uniformity of mechanically sown wheat with UAV RGB imagery. Remote Sens. 9 (12), 1241. doi: 10.3390/rs9121241

[B154] Livingston G. Schonberger S. Delaney S. (2011). Sub-Saharan Africa: The state of smallholders in agriculture. Paper presented at the Conference on New Directions for Smallholder Agriculture. (Rome: International Fund for Agricultural Development (IFAD)) 24, 25.

[B156] Lorence A. Jimenez K. M. (Eds.) (2022). High-throughput plant phenotyping: Methods and protocols Vol. 2539 (New York, USA: Springer Nature). Available at: 10.1007/978-1-0716-2537-8

[B157] Mahdy E. M. B. Ahmad H. (2023). A study of Corchorus L. diversity in Egypt using high-throughput phenotyping platform (HTPP): an Egyptian gene bank example. Genet. Resour. Crop Evolution. 70, 1–11. doi: 10.1007/s10722-023-01551-6

[B158] Maimaitijiang M. Ghulam A. Sidike P. Hartling S. Maimaitiyiming M. Peterson K.. (2017). Unmanned Aerial System (UAS)-based phenotyping of soybean using multi-sensor data fusion and extreme learning machine. ISPRS J. Photogrammetry Remote Sens. 134, 43–58. doi: 10.1016/j.isprsjprs.2017.10.011

[B159] Manzi H. Gweyi-Onyango J. P. (2021). “Agro-ecological lower midland zones IV and V in Kenya using GIS and remote sensing for climate-smart crop management,” in African handbook of climate change adaptation. Eds. Oguge N. Ayal D. Adeleke L. da Silva I. (Cham: Springer). doi: 10.1007/978-3-030-45106-6_35

[B160] Matsumoto T. Wu J. Kanamori H. Katayose Y. Fujisawa M. Namiki N.. (2005). The map-based sequence of the rice genome. Nature 436 (7052), 793–800. doi: 10.1038/nature03895 16100779

[B161] Mbaye M. Ndour A. Gano B. Dembele J. S. B. Luquet D. Beurier G.. (2022). “UAV method based on multispectral imaging for field phenotyping,” in Crop adaptation and improvement for drought-prone environments. Eds. Kane N. A. Daniel K. Dalton T. J. (Manhattan: New Prairie Press), 173–187, ISBN: ISBN 978-1-944548-46-9.

[B162] Meliho M. Khattabi A. Jobbins G. Sghir F. (2020). Impact of meteorological drought on agriculture in the Tensift watershed of Morocco. J. Water Climate Change 11 (4), 1323–1338. doi: 10.2166/wcc.2019.279

[B163] Mengistu D. K. Kidane Y. G. Fadda C. Pè M. E. (2018). Genetic diversity in Ethiopian durum wheat (Triticum turgidum var durum) inferred from phenotypic variations. Plant Genet. Resour. 16 (1), 39–49. doi: 10.1017/S1479262116000393

[B164] Minervini M. Scharr H. Tsaftaris S. A. (2015). ). Image analysis: The new bottleneck in plant phenotyping [applications corner]. IEEE Signal Process. Magazine 32 (4), 126–131. doi: 10.1109/MSP.2015.2405111

[B165] Mukhawana M. B. Kanyerere T. Kahler D. (2023). Review of in-Situ and remote sensing-based indices and their Applicability for integrated drought monitoring in South Africa. Water 15 (2), 240. doi: 10.3390/w15020240

[B167] Müller-Linow M. Pinto-Espinosa F. Scharr H. Rascher U. (2015). The leaf angle distribution of natural plant populations: Assessing the canopy with a novel software tool. Plant Methods 11 (1), 1–16. doi: 10.1186/s13007-015-0052-z 25774205 PMC4359433

[B168] Munghemezulu C. Mashaba-Munghemezulu Z. Ratshiedana P. E. Economon E. Chirima G. Sibanda S. (2023). Unmanned aerial vehicle (UAV) and spectral datasets in South Africa for precision agriculture. Data 8 (6), 98. doi: 10.3390/data8060098

[B169] Musungwini S. (2018). Mobile phone use by Zimbabwean smallholder farmers: A baseline study. Afr. J. Inf. Communication 22, 29–52. doi: 10.23962/10539/26171

[B170] Mutanga O. Dube T. Ahmed F. (2016). Progress in remote sensing: vegetation monitoring in South Africa. South Afr. Geographical J. 98 (3), 461–471. doi: 10.1080/03736245.2016.1208586

[B172] Neilson E. H. Edwards A. M. Blomstedt C. K. Berger B. Møller B. L. Gleadow R. M. (2015). Utilization of a high-throughput shoot imaging system to examine the dynamic phenotypic responses of a C4 cereal crop plant to nitrogen and water deficiency over time. J. Exp. Bot. 66 (7), 1817–1832. doi: 10.1093/jxb/eru526 25697789 PMC4378625

[B173] Ninomiya S. (2022). High-throughput field crop phenotyping: current status and challenges. Breed. Sci. 72 (1), 3–18. doi: 10.1270/jsbbs.21069 36045897 PMC8987842

[B174] Nyaga J. M. Onyango C. M. Wetterlind J. Söderström M. (2021). Precision agriculture research in sub-Saharan Africa countries: A systematic map. Precis. Agric. 22, 1217–1236. doi: 10.1007/s11119-020-09780-w

[B175] Ochieng J. Knerr B. Owuor G. Ouma E. (2020). Food crops commercialization and household livelihoods: Evidence from rural regions in Central Africa. Agribusiness 36 (2), 318–338. doi: 10.1002/agr.21619

[B176] Ogutu G. E. O. Franssen W. H. P. Supit I. Omondi P. Hutjes R. W. A. (2018). Probabilistic maize yield prediction over East Africa using dynamic ensemble seasonal climate forecasts. Agric. For. Meteorology 250–251, 243–261. doi: 10.1016/j.agrformet.2017.12.256

[B178] Osiemo J. Girvetz E. H. Hasiner E. Schroeder K. Treguer D. Juergenliemk A.. (2021). Digital agriculture profile: Kenya (Rome: FAO). Available at: https://www.fao.org/3/cb3958en/cb3958en.pdf (Accessed August 8, 2023).

[B179] Ouraich I. Tyner W. E. (2018). Moroccan agriculture, climate change, and the Moroccan Green Plan: A CGE analysis. Afr. J. Agric. Resource Economics 13 (311-2019-681), 307–330. doi: 10.22004/ag.econ.284990

[B180] Pajares G. (2015). Overview and current status of remote sensing applications based on unmanned aerial vehicles (UAVs). Photogrammetric Eng. Remote Sens. 81 (4), 281–330. doi: 10.14358/PERS.81.4.281

[B181] Palmer P. I. Wainwright C. M. Dong B. Maidment R. I. Wheeler K. G. Gedney N.. (2023). Drivers and impacts of Eastern African rainfall variability. Nat. Rev. Earth Environ. 4 (4), 254–270. doi: 10.1038/s43017-023-00397-x

[B182] Park S. Ryu D. Fuentes S. Chung H. Hernández-Montes E. O’Connell M. (2017). Adaptive estimation of crop water stress in nectarine and peach orchards using high-resolution imagery from an unmanned aerial vehicle (UAV). Remote Sens. 9 (8), 828. doi: 10.3390/rs9080828

[B183] Parkes B. DeFrance D. Sultan B. Ciais P. Wang X. (2018). Projected changes in crop yield mean and variability over West Africa in a world 1.5K warmer than the pre-industrial era. Earth System Dynamics 9 (1), 119–134. doi: 10.5194/esd-9-119-2018

[B184] Parks S. E. Irving D. E. Milham P. J. (2012). A critical evaluation of on-farm rapid tests for measuring nitrate in leafy vegetables. Scientia Hortic. 134, 1–6. doi: 10.1016/j.scienta.2011.10.015

[B185] Parwada C. Marufu H. (2023). Digitalisation of agriculture in Zimbabwe: Challenges and opportunities. Int. J. Sustain. Agric. Res. 10 (1), 32–41. doi: 10.18488/ijsar.v10i1.3280

[B186] Paterson A. H. Bowers J. E. Bruggmann R. Dubchak I. Grimwood J. Gundlach H.. (2009). The Sorghum bicolor genome and the diversification of grasses. Nature 457 (7229), 551–556. doi: 10.1038/nature07723 19189423

[B187] Pauli D. Andrade-Sanchez P. Carmo-Silva A. E. Gazave E. French A. N. Heun J.. (2016). Field-based high-throughput plant phenotyping reveals the temporal patterns of quantitative trait loci associated with stress-responsive traits in cotton. G3: Genes Genomes Genet. 6 (4), 865–879. doi: 10.1534/g3.115.023515 PMC4825657 26818078

[B189] Peng Y. Gitelson A. A. Keydan G. Rundquist D. C. Moses W. (2011). Remote estimation of gross primary production in maize and support for a new paradigm based on total crop chlorophyll content. Remote Sens. Environ. 115 (4), 978–989. doi: 10.1016/j.rse.2010.12.001

[B188] Peng X. Han W. Ao J. Wang Y. (2021). Assimilation of LAI derived from UAV multispectral data into the SAFY model to estimate maize yield. Remote Sens. 13 (6), 1094. doi: 10.3390/rs13061094

[B191] Pieruschka R. Schurr U. (2019). Plant phenotyping: Past, present, and future. Plant Phenomics 2019. doi: 10.34133/2019/7507131 PMC7718630 33313536

[B190] Pieruschka R. Schurr U. (2022). “Origins and drivers of crop phenotyping,” in Advances in plant phenotyping for more sustainable crop production. Ed. Walter A. (Cambridge, UK: Burleigh Dodds Science Publishing Limited), 1–25.

[B192] Pineda M. Barón M. Pérez-Bueno M. L. (2021). Thermal imaging for plant stress detection and phenotyping. Remote Sens. 13 (1), 68. doi: 10.3390/rs13010068

[B193] Poblete T. Ortega-Farías S. Ryu D. (2018). Automatic co-registration algorithm to remove canopy shaded pixels in UAV-borne thermal images to improve the estimation of crop water stress index of a drip-irrigated cabernet sauvignon vineyard. Sensors (Switzerland) 18 (2), 397. doi: 10.3390/s18020397 PMC5856051 29385722

[B194] Potgieter A. B. Watson J. Eldridge M. Laws K. George-Jaeggli B. Hunt C.. (2018). Determining crop growth dynamics in sorghum breeding trials through remote and proximal sensing technologies. International Geoscience and Remote Sensing Symposium (IGARSS), Valencia, Spain. (Piscataway, New Jersey, United States: The Institute of Electrical and Electronics Engineers (IEEE)). doi: 10.1109/IGARSS.2018.8519296

[B195] Qiu Q. Sun N. Bai H. Wang N. Fan Z. Wang Y.. (2019). Field-based high-throughput phenotyping for maize plant using 3d LIDAR point cloud generated with a “phenomobile”. Front. Plant Sci. 10. doi: 10.3389/fpls.2019.00554 PMC6514377 31134110

[B196] Qiu R. Wei S. Zhang M. Li H. Sun H. Liu G.. (2018). Sensors for measuring plant phenotyping: A review. Int. J. Agric. Biol. Eng. 11 (2), 1–17. doi: 10.25165/j.ijabe.20181102.2696

[B197] Quahir. (2022). Drought research in the dry areas of Africa: the case of PhenoMA the high throughput phenotyping Platform in Morocco. Available at: https://www.plantphenotyping.org/lw_resource/datapool/systemfiles/elements/files/0dec546e-673e-11ed-9086-dead53a91d31/current/document/abstract_Book_IPPS_2022.pdf.

[B198] Quemada M. Gabriel J. L. Zarco-Tejada P. (2014). Airborne hyperspectral images and ground-level optical sensors as assessment tools for maize nitrogen fertilization. Remote Sens. 6 (4), 2940–2962. doi: 10.3390/rs6042940

[B199] Rahaman M. M. Chen D. Gillani Z. Klukas C. Chen M. (2015). Advanced phenotyping and phenotype data analysis for the study of plant growth and development. Front. Plant Sci. 6. doi: 10.3389/fpls.2015.00619 PMC4530591 26322060

[B200] Ray D. K. Ramankutty N. Mueller N. D. West P. C. Foley J. A. (2012). Recent patterns of crop yield growth and stagnation. Nat. Commun. 3, 1293. doi: 10.1038/ncomms2296 23250423

[B201] Rebetzke G. J. Fischer R. A. Van Herwaarden A. F. Bonnett D. G. Chenu K. Rattey A. R.. (2014). Plot size matters: Interference from intergenotypic competition in plant phenotyping studies. Funct. Plant Biol. 41 (2), 107–118. doi: 10.1071/FP13177 32480971

[B202] Reynolds D. Baret F. Welcker C. Bostrom A. Ball J. Cellini F.. (2019). What is cost-efficient phenotyping? Optimizing costs for different scenarios. Plant Sci. 282, 14–22. doi: 10.1016/j.plantsci.2018.06.015 31003607

[B203] Reynolds M. Chapman S. Crespo-Herrera L. Molero G. Mondal S. Pequeno D. N. L.. (2020). Breeder friendly phenotyping. Plant Sci. 295, 110396. doi: 10.1016/j.plantsci.2019.110396 32534615

[B204] Rezende W. S. Beyene Y. Mugo S. Ndou E. Gowda M. Sserumaga J. P.. (2020). Performance and yield stability of maize hybrids in stress-prone environments in eastern Africa. Crop J. 8 (1), 107–118. doi: 10.1016/j.cj.2019.08.001

[B205] Roitsch T. Cabrera-Bosquet L. Fournier A. Ghamkhar K. Jiménez-Berni J. Pinto F.. (2019). Review: New sensors and data-driven approaches—A path to next generation phenomics. Plant Sci. 282, 2–10. doi: 10.1016/j.plantsci.2019.01.011 31003608 PMC6483971

[B206] Rosenqvist E. Großkinsky D. K. Ottosen C. O. van de Zedde R. (2019). The phenotyping dilemma—the challenges of a diversified phenotyping community. Front. Plant Sci. 10. doi: 10.3389/fpls.2019.00163 PMC6403123 30873188

[B207] Roudier P. Sultan B. Quirion P. Berg A. (2011). The impact of future climate change on West African crop yields: What does the recent literature say? Global Environ. Change 21 (3), 1073–1083. doi: 10.1016/j.gloenvcha.2011.04.007

[B208] Sadeghi-Tehran P. Virlet N. Hawkesford M. J. (2021). A neural network method for classification of sunlit and shaded components of wheat canopies in the field using high-resolution hyperspectral imagery. Remote Sens. 13 (5), 898. doi: 10.3390/rs13050898

[B209] Sagan V. Maimaitijiang M. Sidike P. Eblimit K. Peterson K. T. Hartling S.. (2019). UAV-based high resolution thermal imaging for vegetation monitoring, and plant phenotyping using ICI 8640 P, FLIR Vue Pro R 640, and thermomap cameras. Remote Sens. 11 (3), 330. doi: 10.3390/rs11030330

[B210] Saidi A. S. Diouri M. (2017). Food self-sufficiency under the Green-Morocco Plan. J. Exp. Biol. Agric. Sci. 5 (Spl-1-SAFSAW), 33–40. doi: 10.18006/2017.5(Spl-1-SAFSAW).S33.S40

[B211] Salami A. Kamara A. B. Schwidrowski Z. B. (2010). Smallholder agriculture in east Africa: Trends, constraints and opportunities (Tunis, Tunisia: African Development Bank).

[B212] Sankaran S. Khot L. R. Espinoza C. Z. Jarolmasjed S. Sathuvalli V. R. Vandemark G. J.. (2015). Low-altitude, high-resolution aerial imaging systems for row and field crop phenotyping: A review. Eur. J. Agron. 70, 112–123. doi: 10.1016/j.eja.2015.07.004

[B214] Sayed S. A. Mahmoud A. S. Farg E. Mohamed A. M. Saleh A. M. AbdelRahman M. A.. (2023). A comparative study of big data use in Egyptian agriculture. J. Electrical Syst. Inf. Technol. 10 (1), 21. doi: 10.1186/s43067-023-00090-5

[B215] Scheben A. Batley J. Edwards D. (2018). Revolution in genotyping platforms for crop improvement. Adv. Biochem. Engineering/Biotechnology 164, 37–52. doi: 10.1007/10_2017_47 29356847

[B216] Schirrmann M. Hamdorf A. Garz A. Ustyuzhanin A. Dammer K. H. (2016). Estimating wheat biomass by combining image clustering with crop height. Comput. Electron. Agric. 121, 374–384. doi: 10.1016/j.compag.2016.01.007

[B217] Schmutz J. Cannon S. B. Schlueter J. Ma J. Mitros T. Nelson W.. (2010). Genome sequence of the palaeopolyploid soybean. Nature 463 (7278), 178–183. doi: 10.1038/nature08670 20075913

[B218] Schnable P. S. Ware D. Fulton R. S. Stein J. C. Wei F. Pasternak S.. (2009). The B73 maize genome: Complexity, diversity, and dynamics. Science 326 (5956), 1112–1115. doi: 10.1126/science.1178534 19965430

[B219] Seiffert U. Bollenbeck F. Mock H. P. Matros A. (2010). Clustering of crop phenotypes by means of hyperspectral signatures using artificial neural networks. 2nd Workshop on Hyperspectral Image and Signal Processing: Evolution in Remote Sensing, WHISPERS 2010 - Workshop Program, Reykjavik, Iceland. (Piscataway, New Jersey, United: The Institute of Electrical and Electronics Engineers (IEEE)), 1–4. doi: 10.1109/WHISPERS.2010.5594947

[B220] Shakoor N. Lee S. Mockler T. C. (2017). High throughput phenotyping to accelerate crop breeding and monitoring of diseases in the field. Curr. Opin. Plant Biol. 38, 184–192. doi: 10.1016/j.pbi.2017.05.006 28738313

[B221] Shi Y. Alex Thomasson J. Murray S. C. Ace Pugh N. Rooney W. L. Shafian S.. (2016). Unmanned aerial vehicles for high-throughput phenotyping and agronomic research. PLoS One 11 (7), e0159781. doi: 10.1371/journal.pone.0159781 27472222 PMC4966954

[B222] Shimeles A. Verdier-Chouchane A. Boly A. (2018). Introduction: Understanding the Challenges of the Agricultural Sector in Sub-Saharan Africa. In: Shimeles A. Verdier-Chouchane A. Boly A. (eds) Building a Resilient and Sustainable Agriculture in Sub-Saharan Africa. (Cham., Switzerland: Palgrave Macmillan), 1–12. doi: 10.1007/978-3-319-76222-7_1

[B223] Shokr M. E. (2020). Environmental Applications of Remote Sensing in Egypt: A Review and an Outlook. In: Elbeih S. Negm A. Kostianoy A. (eds) Environmental Remote Sensing in Egypt. (Cham, Switzerland: Springer Geophysics. Springer), 95–126. doi: 10.1007/978-3-030-39593-3_4

[B224] Shonhe T. Scoones I. (2022). Private and state-led contract farming in Zimbabwe: Accumulation, social differentiation and rural politics. J. Agrarian Change 22 (1), 118–138. doi: 10.1111/joac.12473

[B225] Sie E. K. Oteng-Frimpong R. Kassim Y. B. Puozaa D. K. Adjebeng-Danquah J. Masawudu A. R.. (2022). RGB-image method enables indirect selection for leaf spot resistance and yield estimation in a groundnut breeding program in Western Africa. Front. Plant Sci. 13. doi: 10.3389/fpls.2022.957061 PMC9387199 35991399

[B227] Smidt H. J. Jokonya O. (2022). Factors affecting digital technology adoption by small-scale farmers in agriculture value chains (AVCs) in South Africa. Inf. Technol. Dev. 28 (3), 558–584. doi: 10.1080/02681102.2021.1975256

[B228] Soullier G. Demont M. Arouna A. Lançon F. Del Villar P. M. (2020). The state of rice value chain upgrading in West Africa. Global Food Secur. 25, 100365. doi: 10.1016/j.gfs.2020.100365 PMC7299077 32566470

[B229] Sultan B. Gaetani M. (2016). Agriculture in West Africa in the twenty-first century: climate change and impacts scenarios, and potential for adaptation. Front. Plant Sci. 7. doi: 10.3389/fpls.2016.01262 PMC5004487 27625660

[B230] Swinfield T. Lindsell J. A. Williams J. V. Harrison R. D. Agustiono Habibi. (2019). Accurate measurement of tropical forest canopy heights and aboveground carbon using structure from motion. Remote Sens. 11 (8), 928. doi: 10.3390/rs11080955

[B231] Tadele Z. (2017). Raising crop productivity in Africa through intensification. Agronomy 7 (1), 22. doi: 10.3390/agronomy7010022

[B232] Tamene L. D. Ashenafi A. (2022). Digital agriculture profile: Ethiopia. Addis Ababa (Ethiopia): Alliance of Biodiversity International and International Center for Tropical Agriculture (CIAT). CIAT publication. 20 p. Available at: https://hdl.handle.net/10568/119309

[B233] Tariq M. Ahmed M. Iqbal P. Fatima Z. Ahmad S. (2020). Crop phenotyping. In: Ahmed M. (eds) Systems modeling (Singapore: Springer), 45–60. doi: 10.1007/978-981-15-4728-7_2

[B234] Tattaris M. Reynolds M. P. Chapman S. C. (2016). A direct comparison of remote sensing approaches for high-throughput phenotyping in plant breeding. Front. Plant Sci. 7 (AUG2016). doi: 10.3389/fpls.2016.01131 PMC4971441 27536304

[B236] Tilling A. K. O’Leary G. J. Ferwerda J. G. Jones S. D. Fitzgerald G. J. Rodriguez D.. (2007). Remote sensing of nitrogen and water stress in wheat. Field Crops Res. 104 (1–3), 77–85. doi: 10.1016/j.fcr.2007.03.023

[B237] Tilman D. Balzer C. Hill J. Befort B. L. (2011). Global food demand and the sustainable intensification of agriculture. Proceedings of the National Academy of Sciences of the United States of America. 108 (50), 20260–20264. doi: 10.1073/pnas.1116437108 PMC3250154 22106295

[B238] Tripodi P. Nicastro N. Pane C. Cammarano D. (2022). Digital applications and artificial intelligence in agriculture toward next-generation plant phenotyping. Crop Pasture Sci. 18. doi: 10.1071/CP21387

[B1000] Van Dijk M. Morley T. Rau M. L. Saghai Y. (2021). A meta-analysis of projected global food demand and population at risk of hunger for the period 2010–2050. Nature Food 2 (7), 494–501. doi: 10.1038/s43016-021-00322-9 37117684

[B239] Van Duivenbooden N. Pala M. Studer C. Bielders C. L. Beukes D. J. (2000). Cropping systems and crop complementarity in dryland agriculture to increase soil water use efficiency: a review. NJAS: Wageningen J. Life Sci. 48 (3), 213–236. doi: 10.1016/S1573-5214(00)80015-9

[B241] Varshney R. K. Bohra A. Yu J. Graner A. Zhang Q. Sorrells M. E. (2021). Designing future crops: Genomics-assisted breeding comes of age. Trends Plant Sci. 26 (6), 631–649. doi: 10.1016/j.tplants.2021.03.010 33893045

[B242] Vergara-Díaz O. Zaman-Allah M. A. Masuka B. Hornero A. Zarco-Tejada P. Prasanna B. M.. (2016). ). A novel remote sensing approach for prediction of maize yield under different conditions of nitrogen fertilization. Front. Plant Sci. 7 (MAY2016). doi: 10.3389/fpls.2016.00666 PMC4870241 27242867

[B243] Verner D. Treguer D. Redwood J. Christensen J. McDonnell R. Elbert C.. (2018). Climate variability, drought, and drought management in Morocco's agricultural sector (Washington, D. C., USA: World BankWorld Bank). Available at: https://openknowledge.worldbank.org/handle/10986/30603.

[B244] Virlet N. Lyra D. H. Hawkesford M. J. (2022). Digital phenotyping and genotype-to-phenotype (G2P) models to predict complex traits in cereal crops. in: Walter A (ed.) Advances in Plant Phenotyping for More Sustainable Crop Production. (Cambridge: Burleigh Dodds Science Publishing), p. 40. doi: 10.19103/AS.2022.0102.12

[B245] Virlet N. Sabermanesh K. Sadeghi-Tehran P. Hawkesford M. J. (2017). Field Scanalyzer: An automated robotic field phenotyping platform for detailed crop monitoring. Funct. Plant Biol. 44 (1), 143–153. doi: 10.1071/FP16163 32480553

[B246] Wahab I. (2020). In-season plot area loss and implications for yield estimation in smallholder rainfed farming systems at the village level in Sub-Saharan Africa. GeoJournal 85 (6), 1553–1572. doi: 10.1007/s10708-019-10039-9

[B247] Wang J. Wu B. Kohnen M. V. Lin D. Yang C. Wang X.. (2021). Classification of rice yield using UAV-based hyperspectral imagery and lodging feature. Plant Phenomics 2021, 9765952. doi: 10.34133/2021/9765952 33851136 PMC8028843

[B248] Watt M. Fiorani F. Usadel B. Rascher U. Muller O. Schurr U. (2020). Phenotyping: New windows into the plant for breeders. Annu. Rev. Plant Biol. 71, 689–712. doi: 10.1146/annurev-arplant-042916-041124 32097567

[B249] Wellington M. (2023). Remote Sensing of Croplands, Crop Productivity, and Water use Efficiency with a Focus on Smallholder Systems in Southern Africa (Doctoral dissertation (Australia: The Australian National University).

[B250] Whalen K. Yuhas C. (2019). Low-cost drone and sensor for agricultural applications on small farms in Tanzania. Available at: https://globalwater.osu.edu/files/Drone-Final-Design-Report-1.pdf (Accessed 4 January 2023).

[B252] White J. W. Andrade-Sanchez P. Gore M. A. Bronson K. F. Coffelt T. A. Conley M. M.. (2012). Field-based phenomics for plant genetics research. Field Crops Res. 133, 101–112. doi: 10.1016/j.fcr.2012.04.003

[B251] White J. W. Conley M. M. (2013). A flexible, low-cost cart for proximal sensing. Crop Sci. 53 (4), 1646–1649. doi: 10.2135/cropsci2013.01.0054

[B253] Woldeyohannes A. B. Accotto C. Desta E. A. Kidane Y. G. Fadda C. Pè M. E.. (2020). Current and projected eco-geographic adaptation and phenotypic diversity of Ethiopian teff (Eragrostis teff) across its cultivation range. Agriculture Ecosyst. Environ. 300, 107020. doi: 10.1016/j.agee.2020.107020

[B254] World Bank (2010). “Global strategy to improve agricultural and rural statistics,” in Economic sector work no. 56719-GLB (Washington DC: The International Bank for Reconstruction and Development / The World Bank).

[B255] Xiao Q. Bai X. Zhang C. He Y. (2022). Advanced high-throughput plant phenotyping techniques for genome-wide association studies: A review. J. advanced Res. 35, 215–230. doi: 10.1016/j.jare.2021.05.002 PMC8721248 35003802

[B256] Xie C. Yang C. (2020). A review on plant high-throughput phenotyping traits using UAV-based sensors. Comput. Electron. Agric. 178, 105731. doi: 10.1016/j.compag.2020.105731

[B261] Yang W. Feng H. Zhang X. Zhang J. Doonan J. H. Batchelor W. D.. (2020). Crop phenomics and high-throughput phenotyping: past decades, current challenges, and future perspectives. Mol. Plant 13 (2), 187–214. doi: 10.1016/j.molp.2020.01.008 31981735

[B258] Yang G. Liu J. Zhao C. Li Z. Huang Y. Yu H.. (2017a). Unmanned aerial vehicle remote sensing for field-based crop phenotyping: Current status and perspectives. Front. Plant Sci. 8. doi: 10.3389/fpls.2017.01111 PMC5492853 28713402

[B263] Yang Z. Shao Y. Li K. Liu Q. Liu L. Brisco B. (2017b). An improved scheme for rice phenology estimation based on time-series multispectral HJ-1A/B and polarimetric RADARSAT-2 data. Remote Sens. Environ. 195. doi: 10.1016/j.rse.2017.04.016

[B259] Yang H. Yang J. Lv Y. He J. (2014). SPAD values and nitrogen nutrition index for the evaluation of rice nitrogen status. Plant Production Sci. 17 (1), 81–92. doi: 10.1626/pps.17.81

[B264] Zaman-Allah M. Vergara O. Araus J. L. Tarekegne A. Magorokosho C. Zarco-Tejada P. J.. (2015). Unmanned aerial platform-based multi-spectral imaging for field phenotyping of maize. Plant Methods 11 (1), 1–10. doi: 10.1186/s13007-015-0078-2 26106438 PMC4477614

[B266] Zendonadi dos Santos N. Piepho H. P. Condorelli G. E. Licieri Groli E. Newcomb M. Ward R.. (2021). High-throughput field phenotyping reveals genetic variation in photosynthetic traits in durum wheat under drought. Plant Cell Environ. 44 (9), 2858–2878. doi: 10.1111/pce.14136 34189744

[B267] Zhang J. Yang C. Song H. Hoffmann W. C. Zhang D. Zhang G. (2016). Evaluation of an airborne remote sensing platform consisting of two consumer-grade cameras for crop identification. Remote Sens. 8 (3), 257. doi: 10.3390/rs8030257

[B270] Zhao C. Zhang Y. Du J. Guo X. Wen W. Gu S.. (2019). Crop phenomics: Current status and perspectives. Front. Plant Sci. 10. doi: 10.3389/fpls.2019.00714 PMC6557228 31214228

[B271] Zhao Y. Zheng B. Chapman S. C. Laws K. George-Jaeggli B. Hammer G. L.. (2021). Detecting sorghum plant and head features from multispectral UAV imagery. Plant Phenomics 2021. doi: 10.34133/2021/9874650 PMC8502246 34676373

[B272] Zhu Y. Yao X. Tian Y. C. Liu X. J. Cao W. X. (2008). Analysis of common canopy vegetation indices for indicating leaf nitrogen accumulations in wheat and rice. Int. J. Appl. Earth Observation Geoinformation 10 (1), 1–10. doi: 10.1016/j.jag.2007.02.006

[B273] Zimbabwe Centre For High Performance Computing. (2021). Zimbabwe centre for high performance computing. Available at: https://zchpc.ac.zw/history/.

